# Multiwavelength Implantable Oximeter for Wireless Monitoring of Tissue Oxygenation in the Presence of Blood Volume Changes

**DOI:** 10.1002/adhm.71623

**Published:** 2026-08-23

**Authors:** Anat Usatinsky, Hugo L. Higueros‐Garcia, Sadi Loai, Samantha Unger, Jacob Trueb, Yonatan Usatinsky, Rawad Alkallas‐Valdiviezo, Noor Abu Jarad, Jonathan Wu, Hai‐Ling M. Cheng, John A. Rogers, Abraham Vázquez‐Guardado, Daniel Franklin

**Affiliations:** ^1^ Institute of Biomedical Engineering University of Toronto Toronto Ontario Canada; ^2^ Ted Rogers Centre for Heart Research Toronto Ontario Canada; ^3^ Querry‐Simpson Institute of Bioelectronics Northwestern University Evanston Illinois USA; ^4^ Faculty of Science and Technology Athabasca University Athabasca Canada; ^5^ The Edward S. Rogers Sr. Department of Electrical and Computer Engineering University of Toronto Toronto Ontario Canada; ^6^ Department of Biomedical Engineering Northwestern University Evanston Illinois USA; ^7^ Department of Mechanical Engineering Northwestern University Evanston Illinois USA; ^8^ Department of Materials Science and Engineering Northwestern University Evanston Illinois USA; ^9^ Department of Neurological Surgery Feinberg School of Medicine Northwestern University Chicago Illinois USA; ^10^ Department of Electrical and Computer Engineering North Carolina State University Raleigh North Carolina USA

**Keywords:** biophotonics, medical implants, multiwavelength, Monte Carlo simulations, tissue oxygenation

## Abstract

Real‐time continuous measurement of tissue oxygenation (StO_2_) can provide insight into organ transplant health and disease progression and provide feedback after medical interventions. However, existing optical oximetry approaches typically assume stable blood volume, limiting their usefulness in real‐world scenarios involving vasoconstriction, vasodilation, or acute blood loss. Here, we leverage miniaturized optical sensors and Bluetooth microprocessors to create a fully implantable oximeter that measures subdermal tissue oxygenation and accounts for varying levels of blood volume. The device utilizes a wireless radiofrequency (RF) power transfer scheme that allows high bandwidth Bluetooth data transmission from three wavelength channels: green (537 nm), red (660 nm), and near‐infrared (NIR) (880 nm). Results show that the addition of a green wavelength improves tissue oxygenation estimation in the presence of varying blood volumes. In silico and in vivo validation of the device and tissue oxygenation algorithm is performed in rats during hypoxia, hypercapnia, and occlusion on a variety of tissues such as paw, subdermal muscle, and kidney. Miniaturized, wireless, battery‐free, implantable devices such as these that support mechanisms to compensate for varying blood volumes for StO_2_ calculations offer the potential for continuous, real‐time, physiologically consistent monitoring of blood hemodynamics under dynamic physiological conditions.

## Graphical abstract

This study presents an implantable, wireless, battery‐free oximeter that can continuously monitor physiologically consistent regional tissue oxygenation under dynamic changes of blood volume. This is achieved through multiwavelength photoplethysmography and validated both via Monte Carlo simulations and in vivo rat studies. Different stress events were performed in the in vivo studies, covering an extensive range of hemodynamic parameters.

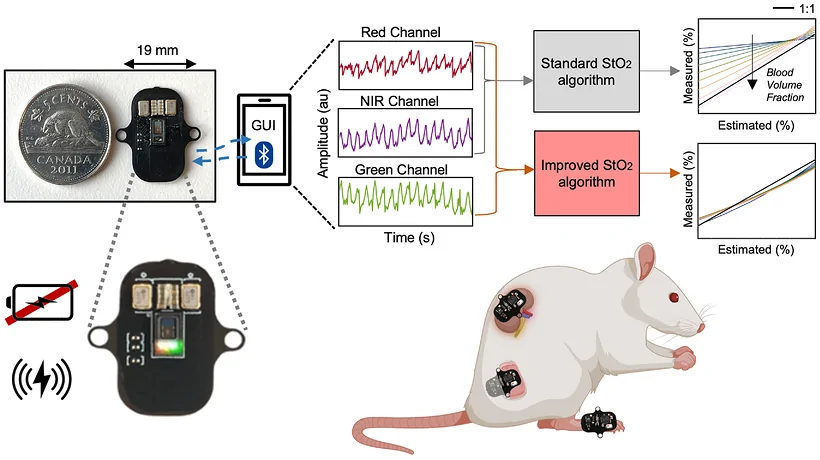

## 1 Introduction

Regional tissue oxygenation (StO_2_) is a key physiological biomarker that relates to cellular processes such as metabolic activity, inflammation, and perfusion [1, 2, 3]. The development of localized real‐time StO_2_ monitoring systems can significantly improve the diagnostics and clinical interventions for conditions, such as sepsis [4, 5], hemorrhagic shock [6, 7], traumatic brain injury [8, 9], organ allotransplantation [10], and acute kidney injury [11, 12]. In addition, there is growing interest in utilizing StO_2_ to remotely monitor conditions that correlate with localized tissue oxygen (O_2_) consumption [13], such as wound healing [14, 15], peripheral arterial disease (PAD) [16, 17], limb ischemia [18, 19], and post‐operative allografts [20, 21].

Current methods of estimating StO_2_ consist of non‐invasive imaging technologies such as blood oxygen level‐dependent magnetic resonance imaging (BOLD MRI) [22], nuclear medicine imaging [23], photoacoustic imaging [24], multispectral imaging [25], and near‐infrared spectroscopy (NIRS) [26]. While these methods can estimate StO_2_ in deep tissues, MRI and nuclear medicine imaging are costly and incompatible for remote and continuous measurements. NIRS and multispectral imaging are rapidly miniaturizing technologies and have been integrated into compact handheld or wearable form factors [27, 28, 29]. However, these light‐based sensing technologies are restricted to near‐surface tissues due to the physical limits of light depth penetration [30, 31]. Photoacoustic StO_2_ can provide spatial oxygenation maps at greater depths than reflectance photoplethysmography (PPG), but quantitative accuracy remains model‐dependent and can be influenced by tissue vascularity, acoustic properties, optical fluence, and anatomy [32]. In contrast, implantable PPG‐based oximetry is better suited for continuous local monitoring from a fixed tissue site.

Alternative techniques are available to enable continuous and remote estimates of StO_2_ within deep tissues (>3 cm). For instance, optical fibers can funnel light to the tissue of interest [33, 34], and optoelectronic components can be integrated into compact implantable probes [35, 36]. Demonstrations include probes for optoelectronic neural modulation [37, 38, 39], photonic probes for calcium ion dynamics [40], hemodynamic catheters [28], and probes for analyzing compartment syndrome [41]. However, the extreme space constraints of these probes (on or below the millimeter scale) limit the types of NIRS techniques that can be implemented, given the source‐detector‐spacing (SDS) is below the optical diffusion approximation of tissue (1–2 cm). Although the differential spectroscopy technique is often applied, it makes assumptions of unchanging blood volume fraction (*f_B_*) in the tissue of interest [42, 43]. The requirement to know blood volume a priori can substantially affect the physiological consistency of StO_2_ when measuring in tissues with unknown disease states, where structural and molecular changes occur [42, 44, 45, 46]. The inability to account for blood volume dynamics limits real‐time StO_2_ monitoring systems to specific tissues under a narrow set of physiological conditions, which exclude ischemia or vascular occlusion, physiological states that play a significant role in conditions like sepsis, hemorrhagic shock, PAD, limb ischemia, and allograft reperfusion injury.

Here, we introduce a battery‐free, wireless implant for hemodynamic monitoring that supports real‐time continuous measurements of tissue oxygenation using three‐wavelength PPG (green: 537 nm; red: 660 nm; NIR: 880 nm) in the presence of blood volume changes. The system incorporates a Bluetooth low energy (BLE) microcontroller (MCU), a custom graphical user interface (GUI), and a power management scheme with a capacitor bank to buffer surges in current consumption during MCU bootup and BLE communication. The use of a modular optical sensor allows dynamic data acquisition of these three wavelength channels and allows full signal digitalization and optimization, on demand, based on the location of implantation and its physiological characteristics over time. We demonstrate device feasibility in rat models undergoing hypercapnic, hypoxic, and occlusive events in tissue beds, including paw, subdermal muscle, and kidney. These protocols and supporting Monte Carlo simulations demonstrate the error associated with classical two‐wavelength (red and NIR), single photodiode, tissue oxygenation models in the presence of blood volume changes and the improvement afforded through incorporation of a volume sensitive [47] third optical channel (green) model developed by Nguyen and colleagues [48]. By providing localized StO_2_ estimates in the presence of blood volume changes, this method could extend the applicability of implantable oximeters to clinical scenarios where soft tissue beds undergo vasoconstriction, vasodilation, or sudden blood loss [49].

## 2 Results and Discussion

**FIGURE 1:**
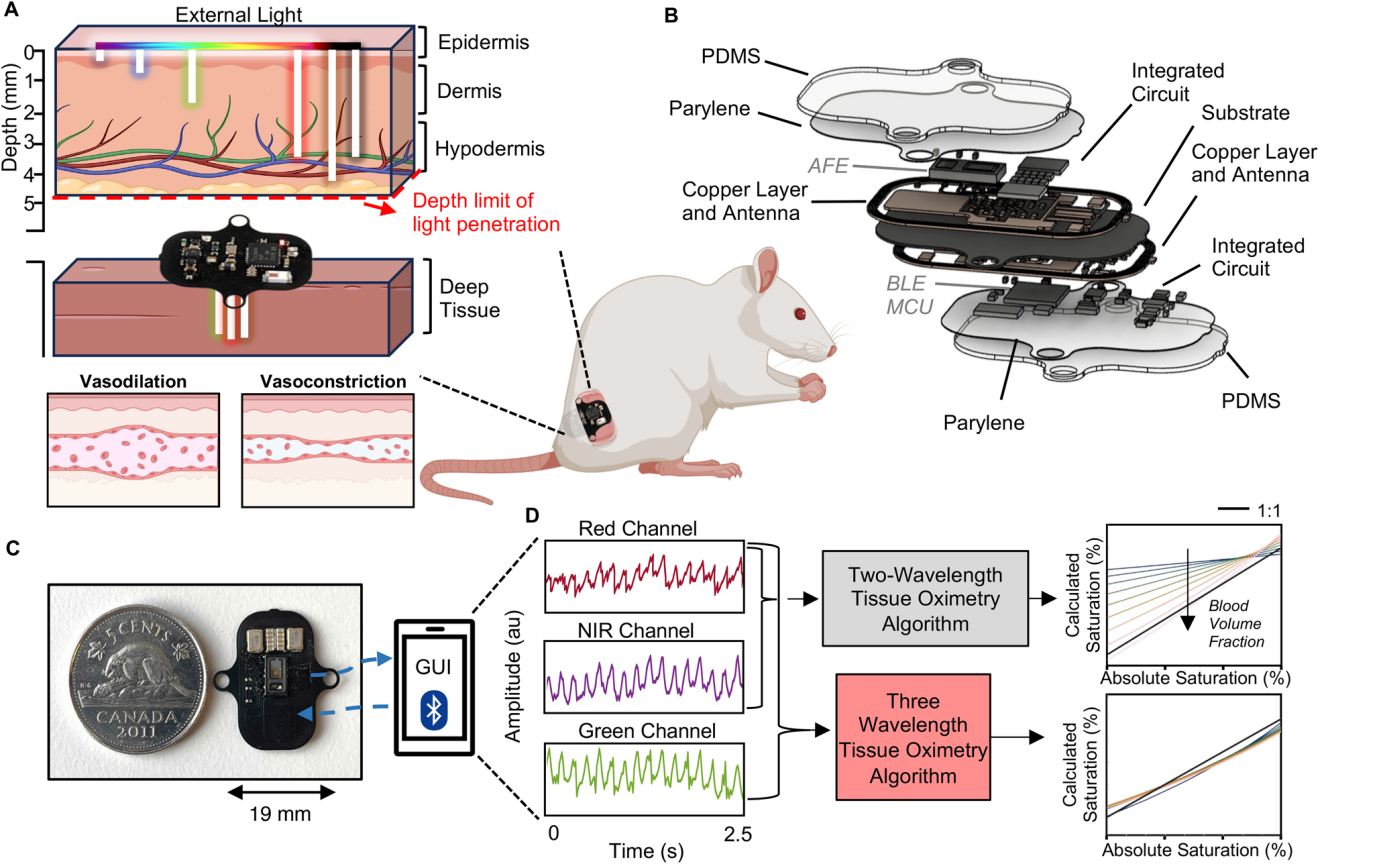
Design of wireless, multi‐wavelength, implantable oximeter. (A) Illustrative representation of the devices being used on a rat model. The green, red, and NIR channels allow for the characterization of blood volume through physiological events such as vasoconstriction and vasodilation below the penetration depth of light at the skin surface. (B) Exploded view illustration of implantable device showcasing its components. (C) Photograph of an implantable device next to a Canadian nickel. (D) Illustrative representation of methods being used for the study. The device communicates with a custom GUI; the blue arrows represent the flow of information. Data acquired from the red and NIR channels is used to calculate tissue oximetry using a two‐wavelength algorithm approach. The data from all channels is used to calculate tissue oximetry using a three‐wavelength algorithm approach. The latter demonstrates better physiological consistency across a variety of blood volume fractions. Illustrations of rat models were created using Biorender.com (https://biorender.com).

The implantation of optoelectronic sensors enables the measurement of subdermal, deep‐tissue hemodynamics or within body cavities, circumventing the limited penetration depth of light in skin tissue (approximately 3.75 mm at 1050 nm) [50] (Figure 1a). Oximeters must function in tissues with unknown blood volumes across dynamic constrictive/dilative states. The multiwavelength oximeter (Figure 1b) consists of a flexible printed circuit board encapsulated in a thin protective conformal coating of Parylene‐C and Polydimethylsiloxane (PDMS) which perform as a water impermeable barrier and mechanically‐matched interface with soft tissues [51], respectively. The compact design of the multiwavelength oximeter (20 mm × 12 mm) enables implantation through small surgical incisions, allowing secured placement of the device on target locations such as muscles and vital organs like the kidneys [35] (Figure 1c). The electronic architecture consists of three subsystems, (i) a modular PPG integrated circuit, (ii) a BLE MCU, and (iii) an inductively coupled power harvesting and management system. The PPG module (MAX30101, Analog Devices) integrates a silicon photodiode (PD), 3 LEDs (green (537 nm), red (660 nm), and NIR (880 nm), and an analog front end (AFE) into a compact package (5.6 mm × 3.3 mm × 1.55 mm) and enables full control of operation parameters: LED intensity, photodiode gain, sampling rates, and sample averaging. The PPG module interfaces with the MCU via Inter‐Integrated Circuit (I^2^C) communication protocol, which configures the module, receives and timestamps optical data, and transmits it to an external GUI via standard BLE protocols (Figure 1d). The custom GUI visualizes and records data sessions in real‐time. It allows the user to update measurement settings of the PPG module. A wireless power harvesting system supports the operation of the PPG module and BLE MCU through inductive magnetic coupling by an external RF coil resonating at 13.56 MHz. Additional information regarding device design, assembly, and the components list is detailed in the supplementary text, Figure S1, and Table S1.

### 2.1 Wireless Power Harvesting and Power Consumption Considerations

The use of wireless power transfer has grown considerably in bioelectronic implantable devices. The device demonstrated here employs wireless power transfer via magnetic induction at RF to power the device, enable battery‐free operation, and achieve a small form factor. Nonetheless, the low induction efficiency between the driving antenna (RF transmitter, two‐coil 60 cm × 60 cm antenna) and implantable wireless device (receiver, four‐coil 19 mm × 10 mm planar antenna), dictated by the physical area of the inductive coils, leads to power harvest limitations that prevent stable operation of high dynamic demands. To mitigate these challenges, a custom power management strategy is implemented for stable operation.

**FIGURE 2:**
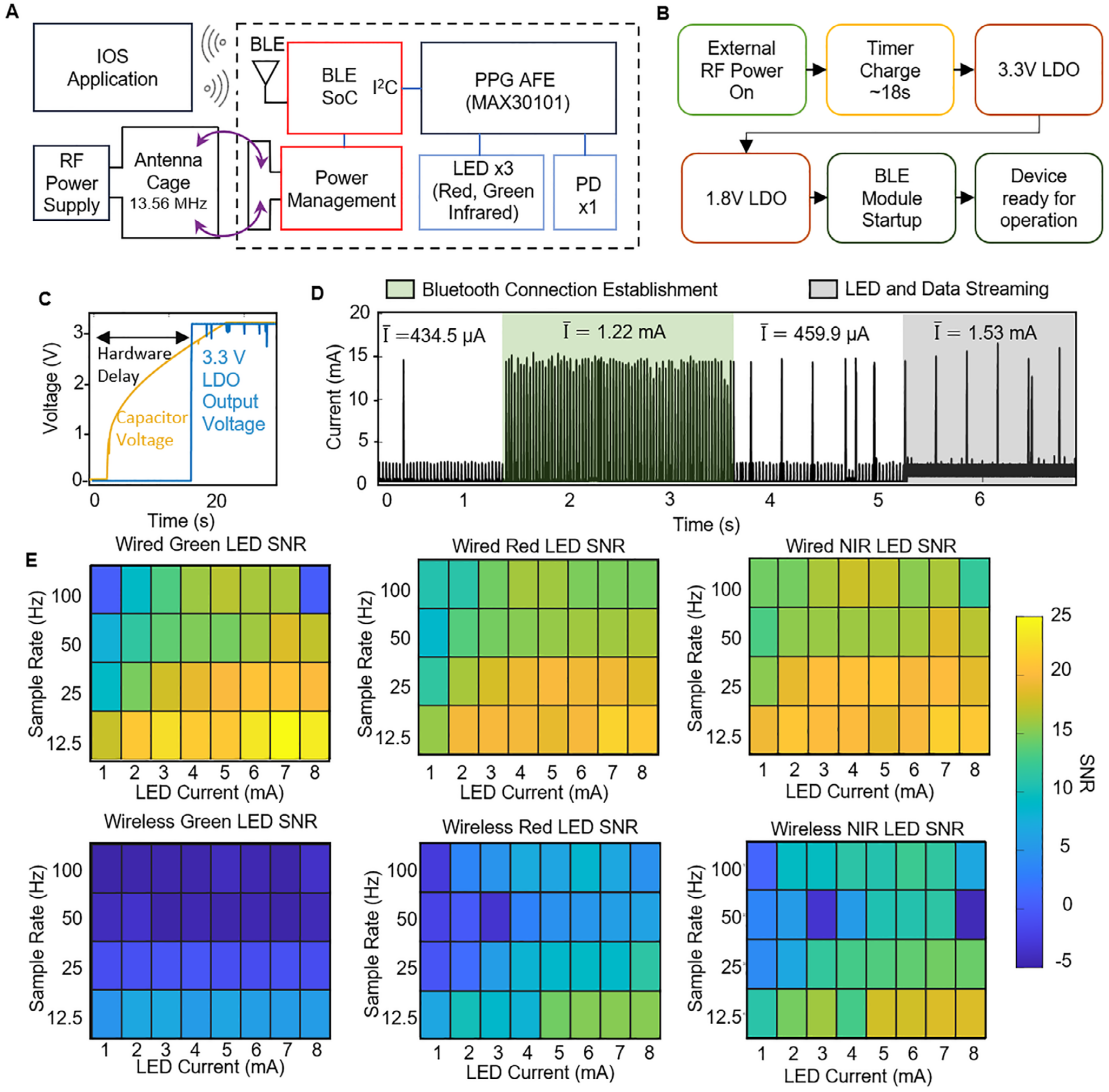
Operational scheme of wireless, implantable oximeter. (A) Operational diagram that includes the RF power supply, the timer charge, LDOs, and the BLE module's advertising mode and operational mode. The dashed outline represents operations that take place inside of the RF setup. Power from the external RF source would flow through the internal antenna, rectified by a pair of parallel capacitors and then through diodes. A comparator was added to allow for the supercapacitors to fully charge and accommodate for the BLE startup before cascading power through an LDO and BLE module. Once connected to an external receiver, the device is ready to start data collection. (B) Boot up the operational diagram. Power from an external source is predicted to flow through the copper antenna, through a 3.3 V LDO, and into the 3.3 V supercapacitors. Afterward BLE modules begin startup, and the device is ready for use. (C) Hardware delay in voltage output due to the average time needed for capacitors to charge to the required voltage. (D) Current consumption of devices under different tasks. Gray box: current consumption during BLE pairing. Light blue box: current consumption during streaming of data. (E) SNR comparison of wired and wireless configurations of implantable devices under different settings. Wired implantable device data was taken with a wired device connected to a Power Profiler Kit II supplied with 3.3 V using the nRF Connect Desktop Power Profiler software (Nordic Semiconductor, Norway). Wireless device data was taken with a wireless implantable device placed in an RF setup.

An external RF power supply operating at 13.56 MHz drives a custom transmitter antenna (19 cm × 27 cm × 16 cm). As illustrated in the operational diagrams (Figure 2a,b), magnetic inductive‐coupling induces an alternating current (AC) signal at 13.56 MHz in the implantable device (the receiver), located inside the perimeter of the transmitter. A half‐wave rectifier formed by two low turn‐on voltage Schottky diodes and a capacitor rectifies the induced AC signal. The first stage of power management consists of a 3.3 V low‐dropout regulator (LDO), which charges a capacitor bank comprised of two supercapacitors (11 mF each) and ten ceramic capacitors (10 µF each) in parallel connection. The high internal resistance of the supercapacitors benefits temporal RF decoupling of the device with the transmitter antenna, whereas the low resistance ceramic capacitor bank allows for fast charge/discharge cycles required by BLE communication. The second stage includes an analog delay implemented with a standard comparator (internal reference of 2.4 V) which monitors the charge stage of the capacitor bank. The output state change of the comparator, ∼18 s after RF power activation (Figure 2c), enables the 1.8 V LDO that powers the electronic device and provides the bootup power surge typical of BLE MCUs (Figure 2d). The power mitigation strategy also reduces the impact of current surges while in operation. When the device is not paired with an external receiver (the BLE advertising stage), the average current consumption is 434.5 µA. During BLE pairing, the average current increases to 1.22 mA and stabilizes at 459.9 µA once the connection is established, and the device enters an idle state. During measurements, the average current increases up to 1.53 mA to accommodate the current demand by the red, NIR, and green LEDs pulsatile operations, as well as the BLE MCU data transfer activities and connection events (Figure 2d).

To show the impact of power harvesting on measurement capabilities and data signal‐to‐noise ratio (SNR), the device is tested under wireless and wired scenarios on a human finger (Figure 2e). Device settings range from 12.5 to 100 Hz and LED current settings of 1 mA to 8 mA for each LED wavelength (green, red, and NIR). The SNR of the raw PPG signals was calculated as the log ratio of the power at the heart rate's fundamental frequency to the integrated noise power above that frequency. For the non‐power‐limited wired devices, SNR increases as the LED current is increased and is only slightly diminished as the sampling rate increases. For the power‐limited wireless device, SNR increases with increasing LED current but substantially decreases for increasing sampling rate, specifically for the green channel with an average SNR of 5.9 dB at 12.5 Hz but −0.89 dB at 25 Hz. Due to the higher forward voltage of the green LED (*V*_F_ = 2.4 V), the green channel consumes more power than the red (*V*_F_ = 1.8 V) and NIR (*V*_F_ = 1.8 V) channels. This can lead to inadequate LED pulses and nosier data. BLE transmission is also highly power intensive, so data drop‐outs can lead to lower SNR for high sampling rates. This data highlights the engineering trade‐off between power consuming tasks and the reduction in power‐harvesting efficiency for miniaturized coils. Idealized settings consist of lower sampling rates of 12.5 or 25 Hz and maximized LED current settings while maintaining a time‐averaged current consumption under 1.5 mA. For detailed insights into the testing setup for verifying the devices' boot‐up and functionality in a wireless environment, refer to Figure S2 of supplementary text. Additional device settings and power testing are also shown in Figure S3 for ADC range, sampling rate, and LED pulse width.

Systematic testing across LED currents and sampling configurations identified an empirical mean‐current threshold of approximately 1.40 mA for the present RF configuration; settings above this threshold produced sporadic channel dropouts, most prominently in the green channel (Figure S4). Propagation of channel‐specific high‐frequency noise through the StO_2_ calculation yielded an estimated resolution limit of approximately 0.1% StO_2_ under stable powering conditions (Figure S5).

Thermal characterization during RF powering and LED operation showed a temperature increase of approximately 2.2°C during RF coupling and less than 1°C with LED activation; device temperatures remained below 43°C under the tested configuration. The absence of a measurable hyperemia‐like response during the initial in vivo activation period further supports negligible thermal perturbation under the conditions studied (Figure S6).

### 2.2 Monte Carlo Simulations of Muscle StO_2_ Under Changing Blood Volume Fraction

**FIGURE 3:**
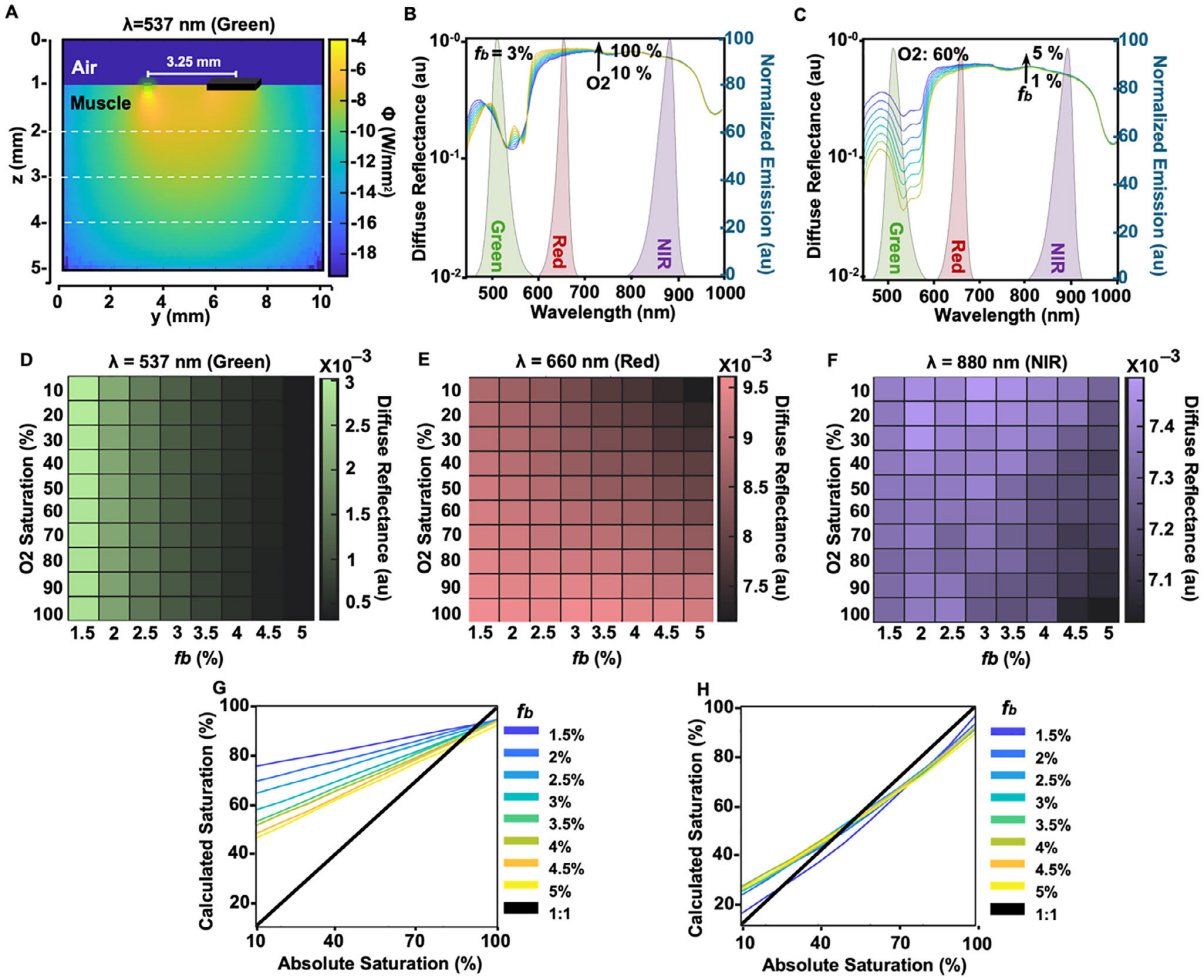
Monte Carlo simulations of tissue oxygenation. (A) Simulation of the flux along the normal direction of the source aperture (*Φ*). The SDS is set to 3.25 mm, matching that of the implantable oximeter. The flux of 537 nm light captured by the photodetector is shown. (B) Reflection spectra of the simulation with a constant *f_B_* and changing blood saturation. Overlapping LED emissions indicate channel sensitivity with reflectance changes. (C) Reflection spectra of the simulation with a constant blood saturation and changing *f_B_*. (D–F) Diffuse reflection spectra heat maps as blood saturation and *f_B_* change for green, red, and NIR wavelengths, respectively. (G) Calculated tissue oxygen with the two‐wavelength model, using red (660 nm) and NIR (880 nm) channels. (H) Calculated tissue oxygen with the model by Nguyen and colleagues, using green (537 nm), red (660 nm), and NIR (880 nm).

To model the performance of the multiwavelength PPG module and compare the accuracy of two‐wavelength and three‐wavelength StO_2_ models, we implement a single‐layer homogenous Monte Carlo model. A single‐layer simulation is selected to align with explicit assumptions of the StO_2_ algorithms and to isolate the effect of *f_B_* on the compared algorithms. Figure 3a demonstrates the flux of light (along the normal of the source aperture) [52] at the green wavelength absorbed by the 1.38 mm × 0.98 mm silicon detector. The SDS of the PPG module is 3.25 mm. Between the emitter and detector, the expected “banana‐shaped” trajectory of detected photons can be observed. The material optical properties can be found in the Methods Section and in the supplementary text, Figure S7. We systematically change the O_2_ and *f_B_* under physiologically relevant values (O_2_ = 10%–100%, *f_B_* = 1%–5%) and simulate diffuse reflectance spectra across 450–1000 nm, calculated from photons absorbed by the virtual photodiode. Figure 3b shows the change of diffuse reflectance due to the variation of oxygen saturation while under a constant *f_B_* value of 3%, while Figure 3c shows the variation of *f_B_* under a constant oxygen saturation of 60%. The morphology and dispersion of the spectral curves for Figure 3b,c agree with experimental results reported in literature for muscle tissue [53, 54, 55, 56]. The three shaded curves illustrate the emission spectra of the LEDs used in the implantable device.

To estimate the impact of oxygen saturation and *f_B_* on each PPG channel, we integrate over the LED emission profiles with the reflectance spectra to obtain a value proportional to the counts reported by the device. Figure 3d–f compile heat maps of the integrated reflectance counts for each of the device wavelength channels. The 537 nm (green) channel, Figure 3d, is highly sensitive to *f_B_*, and largely independent of oxygen saturation. This behavior is consistent with the optical properties of oxy‐ and deoxyhemoglobin (as observed in Figure S7): (1) the optical absorption coefficient at green is more than one order of magnitude than the red and NIR channels; and (2) the green channel is located around the isosbestic point of the optical absorption spectra. Figure 3e shows the 660 nm (red) channel, which has higher absorption with decreasing oxygen saturation and increasing *f_B_* conditions. For the 880 nm (NIR) channel, increased absorption is observed under increasing oxygen saturation and decreasing absorption with increasing *f_B_* conditions. Together, this demonstrates that the green channel has a high correlation to blood volume, independent of blood saturation—whereas red and NIR channels are equally impacted by changes in blood volume and saturation.

In order to compare the expected physiological trends of StO_2_ calculation models, Figure 3g,h illustrate the predicted versus measured StO_2_ results across varying *f_B_* values using the conventional two‐wavelength model [57, 58] and the model by Nguyen and colleagues [48], respectively. The conventional two‐wavelength model introduces an StO_2_ overestimation error under the 10%–80% range and an underestimation at values approaching 100% (Figure 3g). The magnitude of the StO_2_ error showed an inverse relationship with *f_B_*, that becomes more pronounced under lower absolute saturation values. A marked trend change in StO_2_ appears after 70% absolute saturation, where StO_2_ with high *f_B_* values present underestimation errors and StO_2_ with low *f_B_* values present overestimation errors (Figure 3g). As shown in Figure 3h, the three‐wavelength model by Nguyen and colleagues outperformed the conventional two‐wavelength model, with an improved agreement between predicted and measured values across the investigated range of *f_B_*. Together, these simulations show that variations in *f_B_* can be accounted for in compact oximeters through the incorporation of a green channel. An assessment of tissue oxygenation over an extended blood volume range (2%–10%) was also performed to account for physiological conditions like venous congestion and hematoma (detailed in Figure S8).

### 2.3 Demonstration of Blood Volume Changes During Venous and Full Limb Occlusion

**FIGURE 4:**
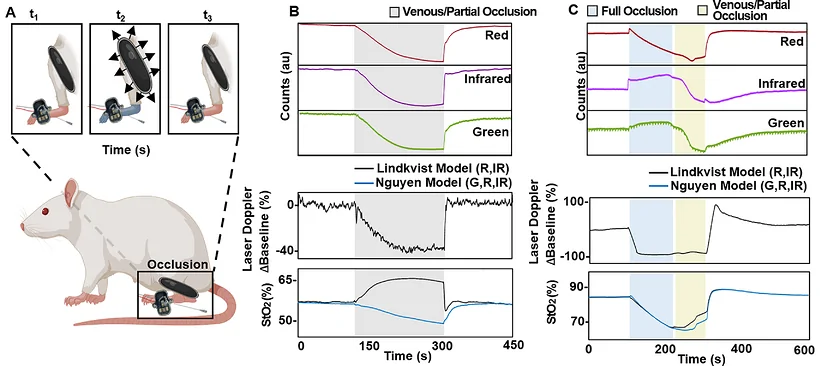
Partial and full occlusion of the hind leg. (A) Scheme of occlusion studies using a 10 mm occlusion cuff (Holly Specialty Products). (B) Data collected from the wired device during the partial, venous occlusion stress study. Raw data collection from each LED channel is depicted on the top panel. Laser Doppler data collection was performed in parallel (Blood FlowMeter, ADInstruments, New Zealand) by placing a probe close to the wired device. Data acquired from the wired device was utilized to calculate StO_2_ under two different models (the two‐wavelength classical model and the Nguyen and colleagues three‐wavelength model). (C) Data collected from the wired device during the partial and full occlusion study. Illustrations of rat models were created using Biorender.com (https://biorender.com).

To demonstrate the impact of large blood volume changes on device performance, a limb occlusion model was implemented in rats. Of the 10 attempted occlusion challenges, six met the inclusion criteria (i.e., low drop counts and absence of data corruption) and were included in the subsequent analysis. A 10 mm occlusion cuff was placed around the hind leg of an anesthetized rat. The oximeter was placed at the hind paw alongside a laser Doppler system for independent confirmation of perfusion trends (Figure 4a), which are highly correlated with blood volume changes (characterized by the “green” channel of the oximeter). Due to the lack of an accepted ‘gold standard’ for tissue oxygenation, laser Doppler flowmetry provides an independent measurement of perfusion trends, whereas the green optical channel is interpreted as a measure sensitive to blood‐volume‐related absorption. These related but non‐equivalent signals are therefore used together to establish the directionality of the occlusion response, rather than as an absolute validation of StO_2_. In equilibrium, the amount of arterial blood entering the limb equals the amount of venous blood leaving the limb. During venous occlusion (Figure 4b), the cuff is partially inflated, which allows oxygenated blood to enter the limb via the arterial system while restricting the outflow of deoxygenated blood through the venous system [59]—causing a build‐up of deoxygenated hemoglobin. This compounds with the ongoing oxygen metabolism within the tissue to further convert oxygenated blood to deoxygenated blood. A gradual decrease in photon counts for the red channel (red line), NIR LED (purple line), and green LED (green line) is observed as the overall blood volume pools during venous occlusion (gray box), consistent with an increase in optical absorption due to an increase in blood volume. The exponentially decreasing flow rate of blood, as measured by laser Doppler, confirms partial occlusion of the limb. At this new equilibrium state, blood volume opposes further blood flow into the limb, while continued metabolic demand further decreases StO_2_. The conventional two‐wavelength oximetry model (black line) predicts a physiologically inconsistent increase and dramatic overestimation of oxygenation. In contrast, the model by Nguyen and colleagues (blue line) captures the expected and physiologically consistent decrease in StO_2_. The maximal StO_2_ difference between the two approaches is 14.5%, with a discrepancy in directionality that can lead to meaningful medical misinterpretation.

Full occlusion (Figure 4c) was performed by fully inflating the occlusion cuff to the maximum recommended setting (150 mmHg), followed by partial occlusion, and then recovery. Under full occlusion, the outflow and inflow of blood in the limb [60] are inhibited, blood volume remains constant, and only variations in oxygenation occur due to the eventual consumption and depletion of available oxygen in blood by adjacent tissues. The red LED (red line) data shows a decline during the full occlusion (blue box) and plateau during the partial occlusion (yellow box). In contrast, the NIR LED (purple line) and green LED (green line) increased during the full occlusion and decreased during the partial occlusion. The laser Doppler confirms the full occlusion and shows a slight restoration of blood flow during partial occlusion. When blood volume remained static during full occlusion, the model developed by Nguyen and colleagues (blue line) and the conventional two‐wavelength model (black line) closely follow each other. However, the two models diverge when partial occlusion allows blood flow into the limb and increases total blood volume. Once the occlusion is released and normal blood flow is restored to the limb, the models again agree. For the occlusion trials, the model by Nguyen and colleagues estimated a mean StO_2_ decrease of 4.25% ± 2.70% (SD) during the stress event, whereas the two‐wavelength model estimated a mean change of 0.02% ± 2.39% (Figure S9).

### 2.4 Demonstration of Subdermal Hypercapnia and Hypoxia Gas Challenges

**FIGURE 5:**
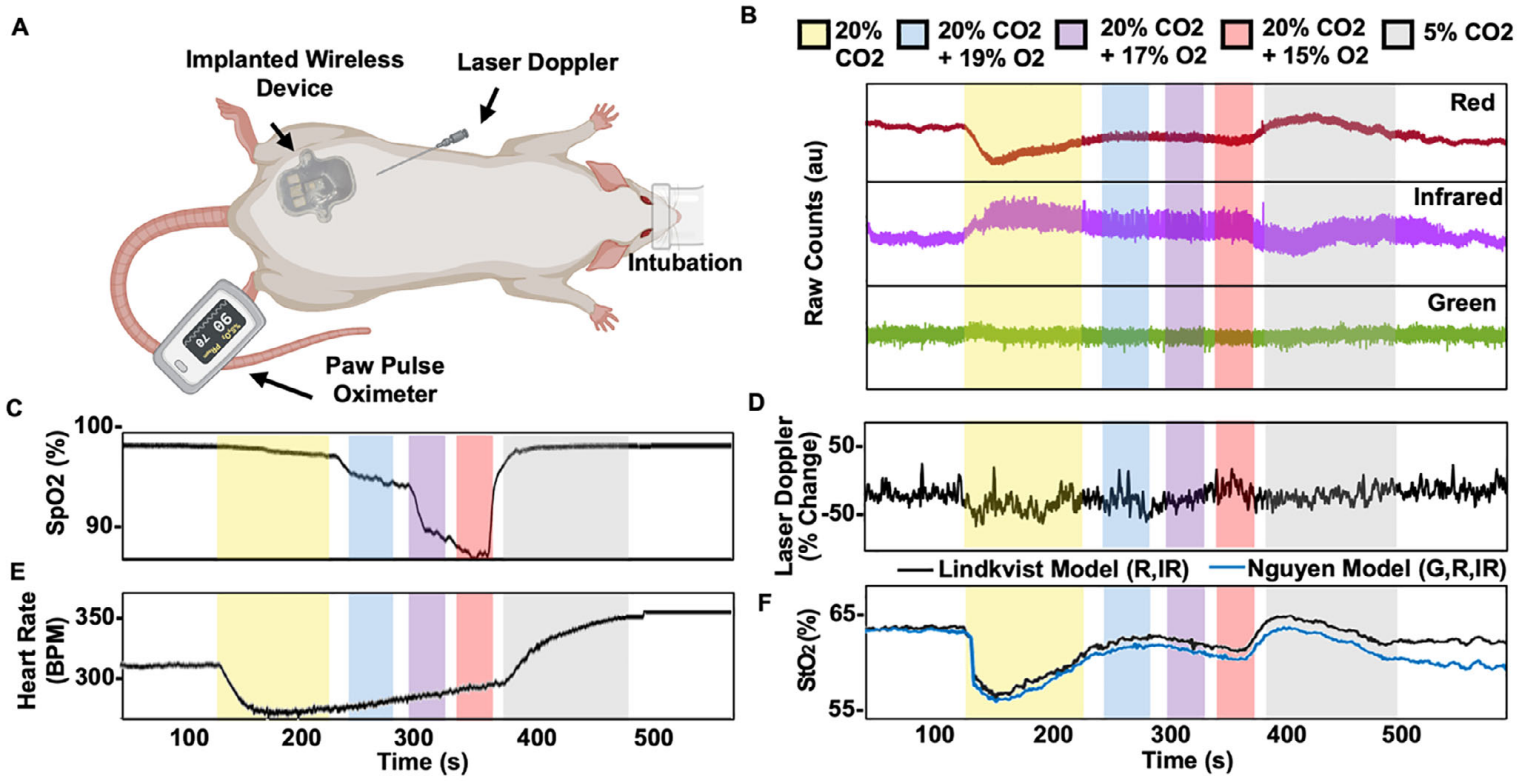
Wireless, subdermal, hypoxia, and hypercapnia stress study. The yellow box indicates when 20% CO_2_ was induced. The **blue** box indicates when 20% CO_2_ + 19% O_2_ was induced. The **purple** box indicates when 20% CO_2_ + 17% O_2_ was induced. The **red** box indicates when 20% CO_2_ + 15% O_2_ was induced. The **gray** box indicates when 50% CO_2_ was induced. (A) Illustration of the experimental set up of the hypoxia and hypercapnia stress study, where rat models were sedated with 2% isoflurane, and placed in an RF setup to power the implantable device. (B) Raw data collection of each LED channel. (C) An oximeter paw clip (MouseOX Plus, Starr Life Sciences, USA) was placed on the paw of the rat to measure SpO_2_. A laser Doppler probe (Blood FlowMeter, ADInstruments, New Zealand) was placed intramuscularly near the implantable device and served to monitor perfusion. (C) Laser Doppler and paw clip oximeter data. (D) Perfusion data collected from the laser Doppler probe during the stress study. (E) Heart rate data captured by the oximeter clip. (F) StO_2_ data of the study calculated using LED channels under two different models (two‐wavelength classical model and the Nguyen and colleagues three‐wavelength model). Illustrations of rat models were created using Biorender.com (https://biorender.com).

To evaluate the functionality of the oximeter in vivo, 4 devices were implanted into a cohort of 4 rats and evaluated over a period of 9 weeks. Of the ten attempted gas challenge protocols in the animal models, six met the inclusion criteria (i.e., low drop‐counts and absence of data corruption) and were included in the subsequent analysis. Figure 5 illustrates the evaluation protocol at Week 8, where inhaled gases (CO_2_ and O_2_) are modified during intubation in the following sequence: room air, 20% CO_2_ (yellow box), 20% CO_2_ + 19% O_2_ (blue box), 20% CO_2_ + 17% O_2_ (purple box), 20% CO_2_ + 15% O_2_ (red box), 5% CO_2_, and back to room air. This sequence is chosen to induce normoxic hypercapnia and progressively increasing levels of hypoxia [61]. Additional information about the functionality tests is described in the supplementary text, Figure S10. The red LED (red line) and NIR LED (purple line) exhibited deviations in photon count from baseline (Figure 5b), reflecting changes in deoxygenated and oxygenated hemoglobin, respectively, consistent with hypoxia. The green LED (green line) did not show appreciable changes from baseline, suggesting that blood volume remained relatively constant throughout the challenge. At the beginning of the sequence (inhaled 20% CO_2_) the model by Nguyen and colleagues estimated a mean StO_2_ decrease of 4.08% ± 2.31% (SD) while the two‐wavelength model estimated a smaller mean StO_2_ decrease of 1.54% ± 1.24% (Figure S9). The pulse oximeter (Figure 5c) demonstrated that peripheral blood oxygen saturation (SpO_2_) and heart rate levels throughout the hypoxia gas challenge decreased in a step wise manner and recovered during the hypercapnia stage, following trends in reported literature [62]. Further supporting this, the laser Doppler (Figure 5d) suggests that throughout the whole gas challenge, blood perfusion levels did not fluctuate from baseline, mimicking the behavior of the green LED channel. When comparing StO_2_ calculations using the model by Nguyen and colleagues (utilizing the green LED data, blue line) and the conventional two‐wavelength model (black line), both models demonstrated similar trends (Figure 5e). This is consistent with the Monte Carlo simulation results, which confirm that both models are appropriate under static blood volume conditions and are robust to changes in arterial saturation.

Examples of weekly testing are shown in Figure S11 and ensure device functionality and quantify signal variability over time, Figure S12. With the implementation of highly functional analog front ends within the PPG module, these weekly variations can be adapted for by changing LED current settings, as shown in Figure S13. After completion of the protocol, histology of tissue surrounding the implanted oximeters confirms the formation of a fibrotic capsule through Masson's Trichrome staining (Figure S14). The ability to dynamically alter oximeter settings in real time enables the adaptation to short‐term and long‐term changing physiological environments. Assessment on tissue oxygenation with fibrotic capsule formation around the device was demonstrated to be robust against measurement bias for the three‐wavelength model by empirically adjusting the fitting parameters (see Supplementary Information, Figure S15).

### 2.5 On‐Organ Tissue Oxygenation Measurements During Hypercapnia

**FIGURE 6:**
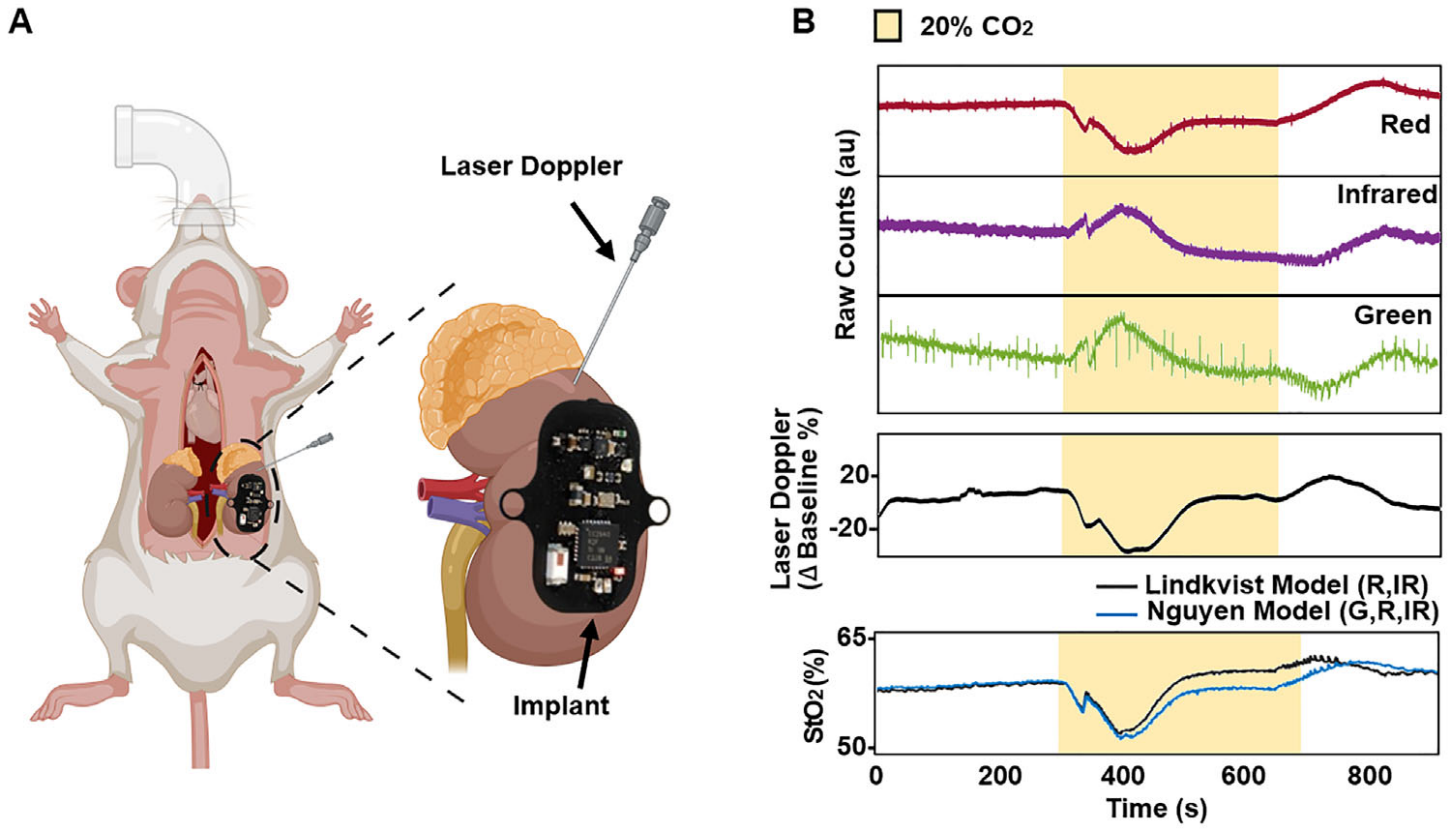
Hypercapnia stress study effects on renal tissue. (A) Scheme of hypercapnia kidney experimental setup. Left: An illustration of an intubated rat on its back with its kidney exposed. Right: The test is set up on the kidney itself. A wireless device is placed on the kidney along with a laser Doppler probe (Blood FlowMeter, ADInstruments, New Zealand). (B) Raw data captured from each LED channel, laser Doppler perfusion percent change, and StO_2_ values calculated with two different models (two‐wavelength classical model and Nguyen and colleagues three‐wavelength model). Illustrations of rat models were created using Biorender.com (https://biorender.com).

Organ tissues exhibit unique vasoconstrictive and vasodilative responses to hypercapnia that differ from muscle tissue [61, 63, 64]. These vasoactive responses induce blood volume changes that can affect StO_2_ estimates. To demonstrate this, the left kidney of two rat models are exposed, and a wireless implantable device is affixed to the kidney, alongside a laser Doppler probe (Figure 6a). Hypercapnia is induced by supplying the rats with 20% CO_2_ (yellow box), allowing the device to assess tissue oxygenation under altered physiological conditions. Real‐time LED data (Figure 6b) collected during the gas challenge show initial changes in photon count across all three LED channels. The red LED (red line) exhibited a decrease below baseline, while the NIR LED (purple line) and green LED (green line) showed an initial increase. All three LEDs returned to baseline during the hypercapnia, likely due to the kidney's compensatory systems [65, 66]. All channels increased again when room air was reintroduced. The laser Doppler (black line) followed an inverse trend to the green LED channel, reflecting dynamic changes in blood flow dynamics. The initial dip in the laser Doppler data suggests a temporary redistribution of blood flow due to system vasoconstriction, followed by compensation to restore blood flow. Then upon recovery, an overcompensation of blood flow occurs, which again equilibrates to baseline. The model by Nguyen and colleagues (blue line) and the two‐wavelength model (black line) exhibit a modest divergence during the inhalation of 20% CO_2_, a difference attributed to the change in blood volume in renal tissue under hypercapnia. The two estimates converge once room air is reintroduced. The model by Nguyen and colleagues estimated a mean StO_2_ decrease of 5.79% during the gas challenge, while the two‐wavelength model estimated a mean decrease of 2.00% (Figure S9).

## 3 Conclusion

This work outlines the development of a wireless, implantable oximeter that estimates StO_2_ in the presence of blood volume changes via a novel three‐wavelength model. Relative to prior two‐wavelength implantable oximeters, the advance evaluated here is a battery‐free, BLE‐enabled three‐wavelength architecture and associated calibration framework that uses the green channel to reduce blood‐volume‐dependent StO_2_ error in a compact single‐detector geometry. A series of in vivo trials were implemented to show functionality during hypercapnia, hypoxia, and occlusion at a variety of physiological locations—designed to capture performance during changes in blood flow, volume, and arterial oxygenation. Monte Carlo simulations confirm behavior observed in vivo, which ranges from a modest shift in estimated StO_2_ for modest blood volume changes, up to physiologically contradictory behavior in scenarios of extreme occlusion. Results show that utilizing the classical two‐wavelength modified Beer‐Lambert models can pose a meaningful, non‐linear variation in StO_2_ trends if *f_B_* deviates from initial assumptions. The differences observed within the hypercapnia experiments are measurable but likely not clinically relevant—in comparison to the occlusion experiment, where the estimated StO_2_ trends are divergent in direction. This suggests that future prioritization of clinical scenarios with large blood volume and perfusion changes—beyond normal physiological vasoactivity—would hold the most benefit from this correction method.

The study has several limitations. Due to the lack of accepted StO_2_ gold‐standards [67], laser Doppler flowmetry was used as an absolute confirmation of perfusion within the tissue beds, rather than as absolute validation of StO_2_. The in vivo results therefore support improved robustness to blood volume variation and physiologically consistent trend detection; independent validation will be required before claims of absolute StO_2_ accuracy or clinical decision thresholds can be established. Future studies could integrate techniques, such as arterial blood gas analysis or pO_2_ sensors, to further validate the findings in this study pertaining to the measurement of StO_2_. Future improvements in wireless power delivery could enable reliable measurement of blood pulsations in vivo, useful for heart rate and pulse oximetry estimations. In addition, a single‐layer Monte Carlo model does not fully reproduce the heterogeneous optical structure of muscle, kidney, paw, or encapsulated tissue. Accordingly, calibration derived for the present device geometry and lean‐muscle parameterization should be re‐established for other tissue beds, altered geometry, or chronic remodeling. Tissue adhesive was used to minimize gross displacement and maintain a consistent sampling location; however, it does not eliminate motion‐induced artifacts. Freely moving studies should incorporate motion‐aware filtering, accelerometry, or artifact‐rejection methods. Lastly, future studies will focus on utilizing reduced transmitter coil dimensions to accommodate translational applications, such as clinical or ambulatory use.

The development of a minimally‐invasive implantable device capable of measuring physiologically consistent StO_2_ under different *f_B_* holds high relevance for the diagnosis and clinical interventions of critical clinical conditions, such as sepsis, hemorrhagic shock, and organ allotransplantation, which all currently lack needed early prognostic indicators, and demonstrates the feasibility of monitoring conditions with diverse blood volume ranges such as wound healing, PAD, limb ischemia, and postoperative allograft care from remote settings. In sepsis, continuous local StO_2_ monitoring could help identify persistent microcirculatory hypoperfusion despite normalization of systemic hemodynamics. In hemorrhagic shock, regional StO_2_ could provide an earlier marker of tissue‐level oxygen debt than systemic blood pressure or heart rate alone [68, 69, 70, 71, 72]. In transplantation, perioperative or early postoperative StO_2_ monitoring could help detect impaired graft perfusion, ischemia‐reperfusion injury, or early allograft dysfunction before conventional serum markers become abnormal [73, 74, 75, 76, 77].

## 4 Materials and Methods

### 4.1 Monte Carlo Simulations to Evaluate Optical StO_2_ Models’ Behavior

Monte Carlo simulations were performed in MATLAB 2023b (MathWorks Inc., USA) using the publicly available light transport simulator, MCXLAB [78] v2019.4. A 3D computational space of 100  × 100  × 50 bins with a total volume of 5 × 10^−1^ cm^3^ used. A total of 1.5 × 10^8^ photon packets were used for each simulation of simulated tissue. An idealized, homogenous, lean muscle layer model was constructed to simulate light propagation within the wavelength window of 450 to 1000 nm with a resolution of 1 nm. The light source, detector, and source detector distance were set up to match the characteristics of the implantable device, with the light source modeled as a coned‐shaped beam (half‐angle of 60 degrees) pointing toward the *z*‐axis. The SDS was set to 3.25 mm. The optical properties of the constituents of the muscle were acquired from literature [42, 79, 80, 81, 82, 83, 84, 85, 86, 87]. Subsequently, a sweep of Monte Carlo simulations was deployed for each combination of *f_B_* and oxygenation saturation values within the reported physiological ranges [83]. The refractive index [87] was assumed to be constant and equal to *n*  =  1.38. Due to the assumption of tissue homogeneity, the absorption coefficient may be calculated as the sum of the individual absorption coefficients [86]:

(1) $$\begin{array}{cc} \mu_{a,tissue}\;\left(\lambda \right)= & f_{B}\;\left(1-O_{2}\right)\mu_{a,Hb}\left(\lambda \right)+f_{B}\cdot O_{2}\cdot \mu_{a,HbO}\left(\lambda \right)\\ & \;+C_{ICW}\cdot \mu_{a,H_{2}O}\left(\lambda \right)+C_{Collagen}\cdot \mu_{a,Collagen}\left(\lambda \right)\\ & \;+C_{Lipids}\cdot \mu_{a,Lipids}\left(\lambda \right)+C_{BT}\cdot \mu_{a,BT}\left(\lambda \right) \end{array}$$

where O_2_ is the true oxygen saturation in muscle, µ_*a*,*Hb*_ is the absorption coefficient of deoxyhemoglobin, µ_*a*, *HbO*_ is the absorption coefficient of oxyhemoglobin, µ_*a*, *Col*_ is the absorption coefficient of collagen, *C_ICW_* is the interstitial water volume reported for muscle, *C_Collagen_* is the concentration for collagen, and *C_Lipids_* is the concentration for lipids, all of which were assumed to be constant. To account for background tissue signal *C_BT_* (concentration of background tissue) and µ_*a*, *BT*_ (fitted absorption coefficient of nonspecific background tissue) were calculated as [86]:

(2) $$\begin{array}{c} \;C_{BT}=1-\left(f_{B}+C_{ICW}+C_{Collagen}+C_{Lipids}\right) \end{array}$$

(3) $$\begin{array}{c} \mu_{a,BT}\;\left(\lambda \right)=\beta \lambda^{-0.26}\;,\;\beta \approx 0 \end{array}$$

The scattering coefficient of the model was calculated as [81]:

(4) $$\begin{array}{cc} \;\mu_{s,tissue}= & f_{B}\;\alpha_{MIE-PY}\left(O_{2}\mu_{s,blood=100\%}\left(\lambda \right)+\left(1-O_{2}\right)\mu_{s,blood=0\%}\left(\lambda \right)\right)\\ & +\frac{\left(1-f_{B}\right)\mu_{s,tissue}\left(\lambda \right)}{1-g_{tissue}\left(\lambda \right)} \end{array}$$

where µ_*s*, *blood*  =  100%_(λ) is the reported scattering coefficient of blood at 100% oxygen saturation, µ_*s*, *blood*  =  0%_(λ) is the reported scattering coefficient of blood at 0% oxygen saturation, *g_tissue_* (λ) is the reported fitted anisotropy factor of muscle as a function of wavelength [87]. The scattering coefficients of blood were rescaled to match the tissue‐specific hematocrit values, calculated via the calculation of the following factor [81]:

(5) $$\begin{array}{c} \;\alpha_{MIE-PY}=\frac{\gamma_{MIE-PY}\left(Hct_{2}\right)\cdot Hct_{2}}{\gamma_{MIE-PY}\left(Hct_{1}\right)\cdot Hct_{1}} \end{array}$$

where *Hct*_1_ is the reported empirically determined hematocrit value, *Hct*_2_ is the actual hematocrit value on the target tissue, and γ_*MIE* − *PY*_(*Hct*) is the hematocrit‐dependent scaling factor function, which can be approximated to: γ_*MIE* − *PY*_(*Hct*) ≈ (1 − *Hct*)^2^. The reduced scattering coefficient was calculated as [86]:

(6) $$\begin{array}{c} \mu_{s,tissue}^{\prime }\;\left(\lambda \right)=\mu_{s,tissue}\;\left(\lambda \right)\left(1-g_{tissue}\left(\lambda \right)\right) \end{array}$$

To calculate the differential pathlength factor (DPF) the following equation was used [88]:

(7) $$\begin{array}{cc} DPF\;\left(\lambda \right)= & \frac{1}{2}\;{\left(\frac{3\mu_{s,tissue}^{\prime }\left(\lambda ,f_{B,0},\;O_{2,0}\;\right)}{\mu_{a,tissue}\left(\lambda ,f_{B,0},\;O_{2,0}\right)}\right)}^{\frac{1}{2}}\\ & \times \left[1-\frac{1}{1-d{\left(\mu_{a,tissue}\left(\lambda ,f_{B,0},\;O_{2,0}\right)\mu_{s,tissue}^{\prime }\left(\lambda ,f_{B,0},\;O_{2,0}\right)\right)}^{\frac{1}{2}}}\right] \end{array}$$

where *d* is the source‐separation distance, *f*_*B*,0_ and *O*_2,0_ are the blood volume fraction and tissue oxygen saturation values reported under normal physiological conditions [83, 85].

### 4.2 Evaluation of Predicted Versus Measured StO_2_ in Lean Muscle Tissue

The resulting simulated diffuse reflectance values were subsequently used to calculate the estimated detected intensity with the following equation:

(8) $$\begin{array}{c} I_{Est}\;\left(\lambda ,f_{B},O_{2}\right)=\int_{\;\lambda_{i}=450}^{\;\lambda_{f}=1000}\xi \left(\lambda ,f_{B},O_{2}\right)\cdot E_{LED}\left(\lambda \right)d\lambda \end{array}$$

where *I_Est_* (λ,*f_B_*, *O*_2_) is the estimated detected intensity for each of the LEDs present on the implant, ξ(λ, *f_B_*, *O*_2_) is the simulated diffuse reflectance calculated by the Monte Carlo simulation model at certain *f_B_* and O_2_, and *E_LED_* is the emission spectrum of the respective commercial LEDs used for the study. The conventional modified Beer‐Lambert law for two‐wavelength approach [58] was used to evaluate the changes in concentration of oxyhemoglobin (Δ[*HbO*]) and deoxyhemoglobin (Δ[*Hb*]):

(9) $$\begin{array}{ccc} & & \left[\begin{array}{c} \Delta \left[Hb\right]\left(f_{B},O_{2}\right)\\ \Delta \left[HbO\right]\left(f_{B},O_{2}\right) \end{array}\right]=\frac{1}{d\cdot f_{B,0}}\\ & & \;\times {\left[\begin{array}{cc} DPF\left(\lambda_{1}\right)\cdot \varepsilon_{Hb}\left(\lambda_{1}\right) & DPF\left(\lambda_{1}\right)\cdot \varepsilon_{HbO}\left(\lambda_{1}\right)\\ DPF\left(\lambda_{2}\right)\cdot \varepsilon_{Hb}\left(\lambda_{2}\right) & DPF\left(\lambda_{2}\right)\cdot \varepsilon_{HbO}\left(\lambda_{2}\right) \end{array}\right]}^{-1}\\ & & \;\times \left[\begin{array}{c} \Delta A\left(\lambda_{1},f_{B},O_{2}\right)\\ \Delta A\left(\lambda_{2},f_{B},O_{2}\right) \end{array}\right] \end{array}$$

where ε_*Hb*_(λ) and ε_*HbO*_(λ) are the molar extinction coefficients of deoxyhemoglobin and oxyhemoglobin, respectively, at a selected wavelength, and Δ*A*(λ, *f_B_*, *O*_2_) is the change in absorbance with respect to each selected wavelength, which is calculated as:

(10) $$\Delta A\;\left(\lambda ,f_{B},O_{2}\right)=\mathrm{lo}g_{10}\left(\frac{\rho \left(\lambda ,f_{B,0},\;O_{2,0}\right)}{\rho \left(\lambda ,f_{B},O_{2}\right)}\right)$$

where ρ(λ, *f_B_*, *O*_2_) is the detected reflectance (ξ or *I_Est_*) at specific *f_B_* and O_2_ values. Predicted reflectance (ξ(λ, *f_B_*, *O*_2_)) and measured reflectance (*I_Est_* (λ,*f_B_*, *O*_2_)) data were subsequently used to independently calculate changes in tissue oxygenation with the following equation:

(11) $$\Delta \mathrm{St}O_{2}\;\left(f_{B},O_{2}\right)=\frac{\Delta \left[\mathrm{HbO}\right]\left(f_{B},O_{2}\right)+\left({\left[\mathrm{Hb}\right]}_{T}\cdot \mathrm{St}O_{2}\left(f_{B,0},O_{2,0}\right)\right)}{\begin{array}{c} \Delta \left[\mathrm{HbO}\right]\left(f_{B},O_{2}\right)+\left({\left[\mathrm{Hb}\right]}_{T}\cdot \mathrm{St}O_{2}\left(f_{B,0},O_{2,0}\right)\right)\\ \;+\Delta \left[\mathrm{Hb}\right]\left(f_{B},O_{2}\right)+\left(1-\mathrm{St}O_{2}\left(f_{B,0},O_{2,0}\right)\right)\cdot {\left[\mathrm{Hb}\right]}_{T} \end{array}}$$

where [*Hb*]_*T*_ is the approximation of total concentration of hemoglobin in tissue, which we set to be [ *Hb*]_*T*_ =  4.5 · 10^−2^ and an initial tissue oxygenation value of *StO*_2_ (*f*_*B*,0_,  *O*_2,0_) =  95% for lean muscle tissue. Nguyen and colleagues’ modified Beer‐Lambert [48] derivation was utilized to calculate StO_2_ for three wavelengths:

(12) $$\mathrm{St}O_{2}\;\left(f_{B},O_{2}\right)=\frac{\begin{array}{c} \mathrm{DPF}\left(\lambda_{3}\right)\log\left(\frac{k_{1}\rho \left(\lambda_{2},f_{B},O_{2}\right)}{\rho \left(\lambda_{1},f_{B},O_{2}\right)}\right)\varepsilon_{\mathrm{Hb}}\left(\lambda_{3}\right)\\ -\mathrm{DPF}\left(\lambda_{1}\right)\log\left(\frac{k_{2}\rho \left(\lambda_{2},f_{B},O_{2}\right)}{\rho \left(\lambda_{3},f_{B},O_{2}\right)}\right)\varepsilon_{\mathrm{Hb}}\left(\lambda_{1}\right)\\ +\mathrm{DPF}\left(\lambda_{2}\right)\log\left(\frac{k_{2}\rho \left(\lambda_{1},f_{B},O_{2}\right)}{k_{1}\rho \left(\lambda_{3},f_{B},O_{2}\right)}\right)\varepsilon_{\mathrm{Hb}}\left(\lambda_{2}\right) \end{array}}{\begin{array}{c} \mathrm{DPF}\left(\lambda_{1}\right)\log\left(\frac{k_{2}\rho \left(\lambda_{2},f_{B},O_{2}\right)}{\rho \left(\lambda_{3},f_{B},O_{2}\right)}\right)\left(\varepsilon_{\mathrm{HbO}}\left(\lambda_{1}\right)-\varepsilon_{\mathrm{Hb}}\left(\lambda_{1}\right)\right)\\ -\mathrm{DPF}\left(\lambda_{3}\right)\log\left(\frac{k_{1}\rho \left(\lambda_{2},f_{B},O_{2}\right)}{\rho \left(\lambda_{1},f_{B},O_{2}\right)}\right)\left(\varepsilon_{\mathrm{HbO}}\left(\lambda_{3}\right)-\varepsilon_{\mathrm{Hb}}\left(\lambda_{3}\right)\right)\\ +\mathrm{DPF}\left(\lambda_{2}\right)\log\left(\frac{k_{1}\rho \left(\lambda_{3},f_{B},O_{2}\right)}{k_{2}\rho \left(\lambda_{1},f_{B},O_{2}\right)}\right)\left(\varepsilon_{\mathrm{HbO}}\left(\lambda_{2}\right)-\varepsilon_{\mathrm{Hb}}\left(\lambda_{2}\right)\right) \end{array}}$$

The fitting parameters *k*_1_ and *k*_2_ depend on device geometry, SDS, tissue optical properties, and tissue‐device interface conditions and were numerically calibrated using Monte Carlo simulations for the present device geometry, selecting values that minimized StO_2_ error across the simulated range of blood volume fractions and oxygen saturations (*k*_1_ = *k*_2_  =  0.77). Because these parameters are not universal constants and vary with the aforementioned factors, recalibration is required if geometry, implantation site, or chronic encapsulation changes. Simulations incorporating a 100 µm collagen layer and an increased SDS of 6.5 mm demonstrate this sensitivity and the capacity for recalibration (Figures S14 and S15).

### 4.3 StO_2_ Real‐Time Implementation on Other Target Tissues

DPF values for renal tissue and paw skin were assumed to be approximately equal to the DPF values calculated for lean muscle. Subsequently, the aforementioned equations for ΔStO_2_ and StO_2_ were adapted to calculate time‐series data obtained from the implantable device as follows:

(13) $$\begin{array}{cc} \left[\begin{array}{c} \Delta \left[Hb\right]\left(t\right)\\ \Delta \left[HbO\right]\left(t\right) \end{array}\right] & =\frac{1}{d\cdot f_{B,0}}\;{\left[\begin{array}{cc} DPF\left(\lambda_{1}\right)\cdot \varepsilon_{Hb}\left(\lambda_{1}\right) & DPF\left(\lambda_{1}\right)\cdot \varepsilon_{HbO}\left(\lambda_{1}\right)\\ DPF\left(\lambda_{2}\right)\cdot \varepsilon_{Hb}\left(\lambda_{2}\right) & DPF\left(\lambda_{2}\right)\cdot \varepsilon_{HbO}\left(\lambda_{2}\right) \end{array}\right]}^{-1}\\ & \;\left[\begin{array}{c} \Delta A\left(\lambda_{1},t\right)\\ \Delta A\left(\lambda_{2},t\right) \end{array}\right] \end{array}$$

(14) $$\Delta A\;\left(\lambda ,t\right)=\mathrm{lo}g_{10}\left(\frac{I_{R}\left(\lambda ,f_{B,0},\;O_{2,0}\right)}{I_{R}\left(\lambda ,t\right)}\right)$$

(15) $$\Delta \mathrm{St}O_{2}\left(t\right)=\frac{\Delta \left[\mathrm{HbO}\right]\left(t\right)+\left({\left[\mathrm{Hb}\right]}_{T}\cdot \mathrm{St}O_{2}\left(f_{B,0},O_{2,0}\right)\right)}{\begin{array}{c} \Delta \left[\mathrm{HbO}\right]\left(t\right)+\left({\left[\mathrm{Hb}\right]}_{T}\cdot \mathrm{St}O_{2}\left(f_{B,0},O_{2,0}\right)\right)\\ \;+\Delta \left[\mathrm{Hb}\right]\left(t\right)+\left(1-\mathrm{St}O_{2}\left(f_{B,0},O_{2,0}\right)\right)\cdot {\left[\mathrm{Hb}\right]}_{T} \end{array}}$$

(16) $$\mathrm{St}O_{2}\;\left(t\right)=\frac{\begin{array}{c} DPF\left(\lambda_{3}\right)\log\left(\frac{k_{1}I_{R}\left(\lambda_{2},t\right)}{I_{R}\left(\lambda_{1},t\right)}\right)\varepsilon_{Hb}\left(\lambda_{3}\right)\\ -DPF\left(\lambda_{1}\right)\log\left(\frac{k_{2}I_{R}\left(\lambda_{2},t\right)}{I_{R}\left(\lambda_{3},t\right)}\right)\varepsilon_{Hb}\left(\lambda_{1}\right)\\ +DPF\left(\lambda_{2}\right)\log\left(\frac{k_{2}I\left(\lambda_{1},t\right)}{k_{1}I\left(\lambda_{3},t\right)}\right)\varepsilon_{Hb}\left(\lambda_{2}\right) \end{array}}{\begin{array}{c} DPF\left(\lambda_{1}\right)\log\left(\frac{k_{2}I_{R}\left(\lambda_{2},t\right)}{I_{R}\left(\lambda_{3},t\right)}\right)\left(\varepsilon_{HbO}\left(\lambda_{1}\right)-\varepsilon_{Hb}\left(\lambda_{1}\right)\right)\\ -DPF\left(\lambda_{3}\right)\log\left(\frac{k_{1}I_{R}\left(\lambda_{2},t\right)}{I_{R}\left(\lambda_{1},t\right)}\right)\left(\varepsilon_{HbO}\left(\lambda_{3}\right)-\varepsilon_{Hb}\left(\lambda_{3}\right)\right)\\ +DPF\left(\lambda_{2}\right)\log\left(\frac{k_{1}I_{R}\left(\lambda_{3},t\right)}{k_{2}I_{R}\left(\lambda_{1},t\right)}\right)\left(\varepsilon_{HbO}\left(\lambda_{2}\right)-\varepsilon_{Hb}\left(\lambda_{2}\right)\right) \end{array}}$$

where *I_R_* is the experimentally recorded signal captured with the implantable device. The effects of SDS on tissue oximetry were evaluated on Supplementary Information, Figure S16.

### 4.4 Fabrication of the Wireless Implantable Device

Flexible printed boards were manufactured by an external supplier (PCBWay, China). The flexible printed circuit thickness was ordered to be 0.1 mm, the length and width were set to 20 mm × 12 mm, and the minimal hole size was set to ≥ φ0.15/0.35 mm. The surface finish was set to Immersion gold (ENIG) (1U″), and the solder mask cover lay was ordered to be a black cover lay. The finished copper was set to 0.5 oz Cu (18 µm). Soldering paste (SMDLTLFPT4, Chip Quik Inc., Canada) bonded the various surface‐mounted components, including the MCU (CC2640R2FRSMT, Texas Instruments, USA), biometric optical sensors (MAX30101EFD+T, Analog Devices, USA), 2.4 GHz Antenna (2450AT18A100E, Johanson Technology Inc., USA), RF Balun (LFB182G45BG5D920, Murata Electronics, Japan), rectifier diodes (RB751S‐40DP‐TP, Micro Commercial Co., USA), and the rest of the components (available in the supplementary text, Table S1) onto the pads by heating at 140°C. Devices were flashed with a hexadecimal source file using a debugging rig contacting the ground, power, TCMS, and TCKS pads connected to a Texas Instruments LAUNCHXL‐CC2640R2 development board utilizing the TI Flash‐Programmer 2 software. The encapsulation process for the wireless module involved chemical vapor deposition (2010 Parylene Coater, Specialty Coating Systems, USA) of parylene‐C with a thickness of 12 µm to yield a Hermetic seal. PDMS (SYLGARD 184 Silicone Elastomer Kit, Dow Corning, USA) was prepared according to the manufacturer instructions in a 10:1 ratio and poured over the assembled PCBs placed inside 3D‐printed molds and cured at 75°C for 2 h. The average weight of the PDMS‐coated implantable device is approximately 1.1 g and is not expected to generate pressures required to induce local ischemic responses. Consistent with this interpretation, baseline recordings from the studied tissues (Figures 4, 5, 6) did not show ischemia‐like behavior. For further detail regarding the implantable device design and assembly, refer to Figure S1 of supplementary text.

### 4.5 RF Setup

The external RF antenna was custom built by wrapping 12 AWG, two‐conductor, parallel silicone wire around a 19 cm × 27 cm × 16 cm plastic box and tuned using a NeuroLux Autotuner (NeuroLux Inc., USA). The antenna's power output was controlled using a high‐frequency, long‐range reader (ID LR5400, FEIG Electronic, USA) via a personal computer.

### 4.6 Measurements of Current Consumed by the Device

The implantable device with wires attached to the ground and power pads prior to encapsulation was used to profile the device's power consumption. The Power Profiler Kit II (PPK2) board (nRF‐PPK2, Nordic Semiconductor, Norway) served as a current measurement tool for the device while connected to a computer with the PPK desktop application. Current consumption thresholds and LED stability were evaluated over different device configurations; for further detail, refer to Note S1 and Figures S4 and S5. The PPK supplied power (a supply voltage of 3.3 V) to the connected device and measured changes in current consumption throughout various device operations.

### 4.7 Measurement of Voltage of Components on the Implantable Device

The implantable device with wires attached to the ground, power pads, and the out pad of the supercapacitors prior to encapsulation was used. Data was collected while a wireless device was placed in the RF setup, and data was collected using LabChart (ADInstruments, New Zealand) and plotted on MATLAB 2023b (MathWorks Inc., USA). Capacitor voltage was collected through the ground and the pad coming out of the super capacitors, and the 3.3 V LDO output voltage was measured using the ground and the power pads.

### 4.8 Animal Preparation

This study was approved by the University of Toronto's Animal Care Committee (protocol ID #20012886). All procedures were conducted in accordance with the Canadian Council on Animal Care. 8‐week male (*n* = 6) and female (*n* = 6) Sprague Dawley rats (Charles River, Canada) were housed three per cage with a 12:12‐h light‐dark cycle and constant room temperature (23°C ± 1°C). All rats were fed a standard chow diet ad libitum for the duration of the study and had free access to water. Male rats weighed between 400 and 500 g and female rats between 200 g and 250 g at the time of implantation and gas challenge.

### 4.9 Surgical Implantation of Wireless Devices In Vivo

Sprague‐Dawley rats of either sex were anesthetized with isoflurane; induced at 5% and then maintained at 1.5%–2% in 1 L min^−1^ oxygen. The depth of anesthesia was monitored for lack of response to a hind limb or tail pinch. Upon failure to elicit a tail or hind limb pinch, rats were positioned on a heat mat to maintain body temperature throughout device implantation. Before incision, rats were injected subcutaneously with meloxicam (2 mg kg^−1^) using a 25G needle. Analgesia was continued every 24 h for a total of three doses (72 h) post‐operatively. The applicable dorsal surface (approximately 1 cm × 1 cm patch on the hind left thigh, front left thigh, dorsal interscapular region, and/or abdomen of the rat) was shaved and cleaned with 0.5% chlorhexidine and 70% alcohol swab. Bupivacaine was used for intradermal injection at the site of incision up to max of 8 mg kg^−1^ using a 27G needle.

The implantable devices were sterilized with PREempt CS20 (Virox Technologies Inc., Canada) for 20 min, according to manufacturer instructions. Device functionality was tested after the cold liquid sterilization prior to implantation by holding them with sterile tweezers inside the RF setup and were observed for their ability to communicate with an external BLE receiver. Up to two small pockets that extended approximately 1.5 cm in total (and no more than 1 cm wide), between the skin and muscle along the spinal column were created in the rats. Care was taken not to damage underlying muscles (pointing the instrument tip up toward the skin). In addition, an optional step was included to allow for testing of wired devices that are prototypes of future device variations. This optional step includes exposing the muscle and placing a sterile and wrapped wired device on it.

Sterilized forceps were used to grasp the implantable device flaps to place the device into the prepared pocket, ensuring that the spectral sensor faced the muscle, lay flat, and that the edge of the device was several millimeters away from the incision site. Care was taken to place the device as parallel to the ground as possible. To glue the implantable devices onto the muscles, Vetbond Tissue Adhesive (1469Sb, 3 M, USA) and Tisseel Prima Fibrin Sealant (Baxter, USA) were used. 4.0–5.0 monofilament non‐absorbable nylon suture was used to close the incision and removed after 14 days. After the end point, the animal models’ samples from the resulting fibrotic capsules formed around the implantable devices were collected and stained for assessment.

### 4.10 Animal Intubation

Animals were anesthetized on 5% isoflurane in room air (2 L min^−1^) and then intubated with a 14–16‐gauge angio‐catheter prior to being transferred to the RF setup. Respiration was maintained at 80 breaths per minute, and animals were kept on a heating pad or under a heat lamp. Isoflurane was maintained at 1.5%–2% throughout the experimental session. Vital signs (heart rate, SpO_2_, respiration rate) of the animal were monitored in real‐time throughout the imaging session using a rodent oximeter (MouseOX Plus, Starr Life Sciences, USA) placed on the hind paw.

### 4.11 Occlusion Trial

Ten separate occlusion trials were attempted in this work, six of which met the inclusion criteria for analysis (scarce drop‐counts and absence of data corruption). Intubated rats, set to 80 breaths per minute, underwent occlusion procedures using an MRI‐compatible ventilator (MRI‐1 Ventilator, CWE, USA) under 1.5%–2.5% isoflurane. A 10 mm occlusion cuff (Holly Specialty Products, USA), inflatable with water, was positioned on the ankle of the rat. An oximeter (MouseOX Plus, Starr Life Sciences, USA) placed on the non‐occluded hind paw monitored SpO_2_, heart rate, and breathing rate. Wired NRG devices, connected to a Power Profiler Kit II (Nordic Semiconductor, Norway) for 3.3 V power, underwent encapsulation with PDMS as described previously and a laser Doppler ((OxyFlo, Oxford Optronix, United Kingdom) or (Blood FlowMeter, ADInstruments, New Zealand)) were placed and secured just below the occlusion site, and tested for cross talk. Following the confirmation of device functionality and calibration, the occlusion was initiated. Data collection of all three devices started at baseline and continued during a 5‐min occlusion activated according to the manufacturer's instructions and persisted post‐cuff release.

### 4.12 Hypoxia and Hypercapnia Gas Challenge

Ten separate gas challenge trials for the study of subdermal tissue were attempted in this work, six of which met the inclusion criteria for analysis (scarce drop‐counts and absence of data corruption). Varying concentrations of CO_2_ were mixed with varying concentrations of O_2_, balanced with nitrogen in a GSM‐3 gas mixer (CWE, USA), and directed to an intubated rat to achieve varying degrees of hypercapnia and hypoxia responses. Respiration was controlled via ventilator (MRI‐1 Ventilator, CWE, USA); vital signs and blood oxygen saturation were monitored via an oximeter (MouseOX Plus, Starr Life Sciences, USA). Blood perfusion at specific sites monitored using a laser Doppler (OxyFlo, Oxford Optronix, United Kingdom, or Blood FlowMeter, ADInstruments, New Zealand) based on availability. The laser Doppler and oximeter data were collected and analyzed using LabChart Reader (ADInstruments, New Zealand).

Intubated rats were placed inside of the unplugged external RF antenna on top of a heating pad. Upon proper positioning of the rat, the RF setup was turned on, device interferences were checked, and adjustments were made to ensure no crosstalk between all the equipment. The following sequence of gas challenges took place to induce and track physiological changes: Baseline measurement with room air, 2 min of 20% CO_2_ and 21% O_2_ (normoxic hypercapnia), 1 min of 20% CO_2_ + 19% O_2_, 1 min of 20% CO_2_ + 17% O_2_, 1 min of 20% CO_2_ + 15% O_2_, 2 min of 5% CO_2_ and 21% O_2_, and recovery with room air (0% CO_2_ and 21% O_2_).

### 4.13 On‐Kidney Trial

Following the gas challenges for the study of subdermal tissue, two intubated rats were selected for the on‐kidney trials. These animals had a recovery period of at least 20 min. They were then positioned prone, and a surgical pocket was created in the abdominal skin to access the kidneys. An oximeter (MouseOX Plus, Starr Life Sciences, USA) was secured on the rat's paw, and a laser Doppler (OxyFlo, Oxford Optronix, United Kingdom) fiber optic probe was inserted 2 mm—3 mm deep inside the kidney cortex, along a wireless implantable device coated in PDMS. After securing the devices to the kidney, the RF setup was turned on, and care was taken to check for cross talk, upon which data collection began. The protocol for the hypercapnia test involved providing an intubated rat, initially with room air to establish a baseline, followed by a 5‐min exposure to 20% CO_2_, and concluding with room air to facilitate recovery.

### 4.14 Statistical Analysis

StO_2_ responses at baseline and during stress events were compared across the occlusion (*n* = 6) and gas (*n* = 6) protocols by time‐window averaging, using the first 50 s of baseline and the 50 s preceding the maximal stress response. Data are presented as mean ± SD unless otherwise stated. To evaluate the presence of implantable‐device‐induced hyperemia, two one‐sided tests (TOST) were implemented to compare the mean percent difference between the first and second minute of each LED channel. A channel response was considered equivalent if its CI (95%) was contained within the equivalence bounds (*Δ* = ±1.5%). All analyses were performed in MATLAB R2023b (The MathWorks Inc., USA).

The paper and supporting information contain all data needed to evaluate conclusions presented in this work. Supporting information is available from [*Wiley Online Library or author*].

## Author Contributions

Concept and study design: Anat Usatinsky, Abraham Vázquez‐Guardado, John A. Rogers, Hai‐Ling. M. Cheng, and Daniel Franklin. Device assembly and testing: Anat Usatinsky, Jonathan Wu, Yonatan Usatinsky, and Hugo L. Higueros. Software development: Jacob Trueb. Monte Carlo simulations: Hugo L. Higueros. Animal trial: Anat Usatinsky, Hugo L. Higueros, Sadi Loai, Samantha Unger, and Noor Abu Jarad. Data Analysis: Anat Usatinsky, Hugo L. Higueros, Samantha Unger, Rawad Alkallas, and Daniel Franklin. Writing – original draft: Anat Usatinsky, Hugo L. Higueros, Noor Abu Jarad, Rawad Alkallas, and Daniel Franklin. Writing – review and editing: all authors.

## Funding

This work was supported by the Canadian Foundation for Innovation/Ontario Research Fund; Award no. 42641. Translational Biology and Engineering Program, Ted Rogers Centre for Heart Research Seed Grant.

## Ethics Statement

Ethical approval for this study was obtained from The University of Toronto Animal Care Committee (UACC) with the following Animal Use Protocol (AUP) ID: 20012886. The study was performed at the Division of Comparative Medicine (Medical Sciences Building, University of Toronto).

## Conflicts of Interest

The authors declare no conflicts of interest.

## Supporting information




**Supporting File**: adhm71623‐sup‐0001‐SuppMat.pdf.

## Data Availability

All the data generated or analyzed for this work are included within the manuscript. Additional information requested with the intent of reanalyzed the data reported in this work is available upon request.
