# Supplementary material for: Multiwavelength Implantable Oximeter for Wireless Monitoring of Tissue Oxygenation in the Presence of Blood Volume Changes
Source: Adv Healthc Mater. 2026 Aug 23;15(35):e71623. doi: 10.1002/adhm.71623 (PMC13586640; doi:10.1002/adhm.71623)
Supplement: Supplementary file 1 [file ADHM-15-0-s001.pdf]

Supporting Information

Multiwavelength Implantable Oximeter for Wireless Monitoring of Tissue Oxygenation in the Presence of Blood Volume Changes

*Anat Usatinsky, Hugo L. Higueros, Sadi Loai, Samantha Unger, Jacob Trueb, Yonatan Usatinsky, Rawad Alkallas, Noor Abu Jarad, Jonathan Wu, Hai-Ling. M. Cheng, John A. Rogers, Abraham Vázquez-Guardado, Daniel Franklin**

A. Usatinsky, H. L. Higueros, S. Loai, S. Unger, R. Alkallas, N. Abu Jarad, J. Wu, H-L. M. Cheng, D. Franklin
Institute of Biomedical Engineering, University of Toronto, 164 College St Room 407, Toronto, Ontario, M5S 3G9, Canada.
E-mail: dan.franklin@utoronto.ca

A. Usatinsky, H. L. Higueros, S. Loai, S. Unger, R. Alkallas, N. Abu Jarad, J. Wu, H-L. M. Cheng, D. Franklin
Ted Rogers Center for Heart Research, 661 University Ave, 14th Floor, Toronto, Ontario, M5G 1M1, Canada

J. Trueb, J. A. Rogers
Querry-Simpson Institute of Bioelectronics, Northwestern University, 2145 Sheridan Road, Evanston, IL, 60208, USA.

Y. Usatinsky
Faculty of Science and Technology, Athabasca University, 1 University Dr, Athabasca, AB T9S 3A3, Canada.

H-L. M. Cheng
The Edward S. Rogers Sr. Department of Electrical and Computer Engineering, University of Toronto, 10 King's College Rd, Toronto, Ontario, M5S 3G8, Canada.

J. A. Rogers
Department of Biomedical Engineering, Northwestern University, Evanston, IL, 60208, USA.

J. A. Rogers
Department of Mechanical Engineering, Northwestern University, Evanston, IL, 60208, USA.

J. A. Rogers
Department of Materials Science and Engineering, Northwestern University, Evanston, IL, 60208, USA.

J. A. Rogers
Department of Neurological Surgery, Feinberg School of Medicine, Northwestern University, Chicago, IL, 60611, USA.

A. Vázquez-Guardado
Department of Electrical and Computer Engineering, North Carolina State University, Raleigh, NC 27606, USA

A. Usatinsky and H. L. Higueros contributed equally to this work.

Correspondence: Daniel Franklin (dan.franklin@utoronto.ca, 661 University Avenue, 14th floor, Toronto, ON, M5G 1M1, Canada).

Note S1. Comparison of Pyroelectric Sensitivity between Flexible PZT and PVDF Sensors

LED-channel stability is evaluated using two complementary analyses: (i) representative baseline recordings under power-sufficient conditions and (ii) systematic testing across LED currents and PPG sampling configurations.

We quantified practical sensitivity to optical-channel noise using the high-frequency noise distribution of each LED channel under stable powering conditions. The full width at half maximum (FWHM) was calculated for each channel and then propagated through the StO_2 calculation by adding channel-specific offsets to the raw signals.

$$FWHM = 2\sqrt{\ln 2}\,\sigma$$

Where σ is the standard deviation of the high frequency noise distribution. The impact of this resolution limit/noise floor can then be transferred to StO_2 resolution limits. Adding a NIR FWHM offset to the raw NIR signal leads to a 0.05% StO_2 change. The same logic was applied and tested to the red and green channels, which showed similar results. We also tested opposing perturbations in the red and NIR channels to confirm that the sign of the StO_2 change followed the expected physiological direction. These results indicate that, within the selected powering and sampling regime, the hardware noise floor is small relative to the StO_2 changes observed during physiological challenges. Additionally, this figure also shows that the minimum StO_2 resolution is ~0.1%. We added this analysis to the Supplementary Information.

To determine settings guidelines for ensuring LED stability, we perform iterative stability testing under varying LED currents and PPG sampling rates. Results are shown in Figure S4, where RSR is raw sample rate, SDIV is sample divider, and SPS is samples per second. We empirically identified a mean-current threshold of approximately 1.40 mA for the present RF coil configuration; settings above this threshold produced sporadic channel dropouts, most prominently in the green channel. We therefore selected operating settings below this instability regime while preserving sufficient SNR.

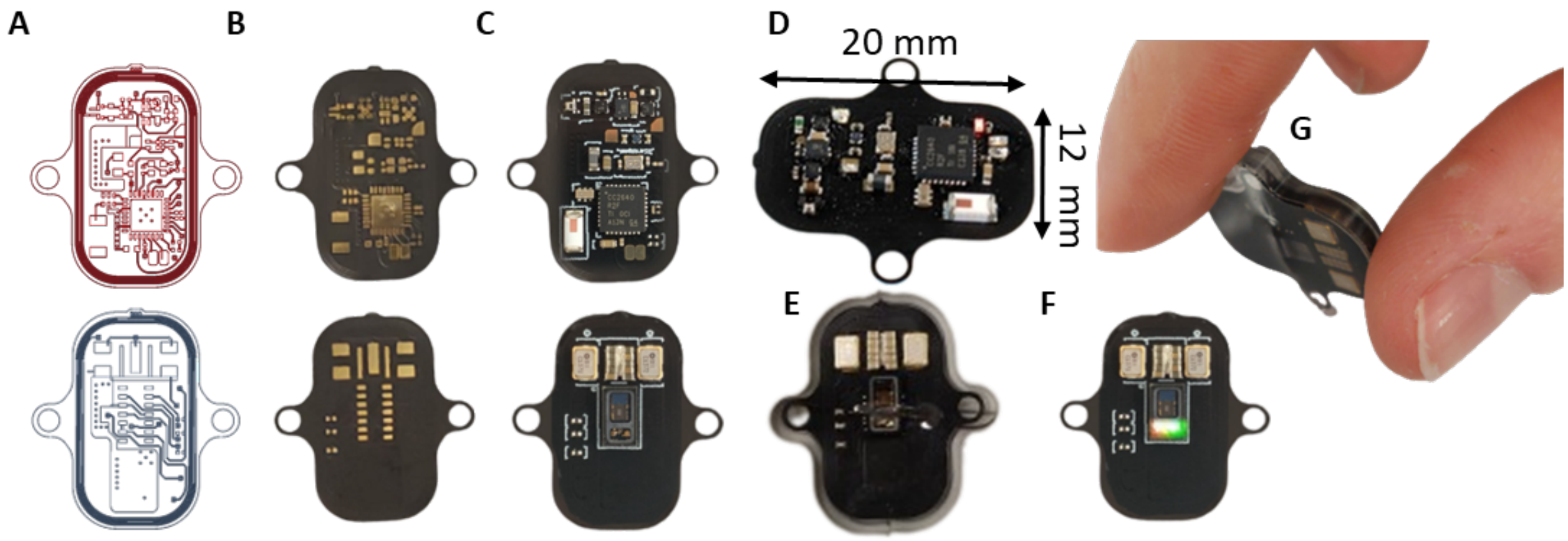

Figure S1. Device design and assembly. A–C. Top row shows the top side of the device; bottom row shows the bottom side of the device. **A.** CAD design of the implantable device. The bottom layer is shown in blue, and the top layer is shown in red. **B.** Blank printed circuit board The flexible printed circuit thickness is 0.1 mm, the length and width are 20 mm x 12 mm. **C.** Assembled Device. Components were soldered onto the PCB. For a comprehensive list, refer to Table S1. **D.** Fully functioning and assembled device and its dimensions. **E.** Complete and assembled device coated in parylene and PDMS. **F.** Device with LED module turned on. **G.** A fully assembled implantable device held by a hand.

Component	Part number	Manufacturer
Schottky diode (30 [mA], 40 [VDC])	RB751S-40DP-TP	Micro Commercial Co
Linear voltage regulator IC (300 [mA])	NCP114AMX330TCG	Onsemi
Linear voltage regulator IC (450 [mA])	NCP161BFCS180T2G	Onsemi
Red LED indicator	APHHS1005SURCK	Kingbright
RF Balun (2.4 [GHz] - 2.5 [GHz], 50 [Ω])	LFB182G45BG5D920	Murata Electronics
Chip RF antenna, surface mount (2.4 [GHz] - 2.5 [GHz])	2450AT18A100E	Johanson Technology Inc
Bluetooth 5.1 Low Energy wireless MCU	CC2640R2FRSMT	Texas Instruments
24 MHz crystal ([MHz], [100Ω])	ECS-240-6-47B-CKM-TR	ECS Inc
IC comparator (1.5 [V] to 5.5 [V], 1.25% reference)	MIC842HYMT-CT-ND	Microchip Technology
Ceramic capacitor (2.2 [μF], 25 [VDC])	GRM155R61E225KE11D	Murata Electronics
Ceramic capacitor (12 [pF], 50 [VDC])	GJM0335C1H120GB01D	Murata Electronics
Ceramic capacitor (10 [μF], 6.3[VDC])	GRM155R60J106ME15D	Murata Electronics
Shielded multilayer inductor (10 [μH], 300 [mA])	LQM18DN100M70L	Murata Electronics
Jumper chip resistor [0Ω]	RK73Z1ERTTP	KOA Speer
Multilayer ceramic capacitor (1 [μF], 6.3 [VDC])	02016D105KAT4A	Kyocera AVX
Chip resistor (10 [kΩ])	AC0201FR-0710KL	YAGEO
Multilayer ceramic capacitor (10 [nF], 10 [VDC])	0201ZC103MAT2A	Kyocera AVX
Chip resistor (100 [kΩ])	CR0201-FW-1003GLF	Bourns
Chip resistor (10 [MΩ])	ERJ-1GNJ106C	Panasonic
Multilayer ceramic capacitor (6 [pF], 25 [VDC])	CBR02C609B3GAC	Kemet
Multilayer ceramic capacitor (47 [pF], 25 [VDC])	C0201C470K3GAC	Kemet
Heart rate monitor and pulse oximeter sensor module	MAX30101EFD+T	Analog Devices
Supercapacitor (11 [mF], 3.3 [VDC])	CPH3225A-2K	Seiko Instruments
Supercapacitor (22 [μF], 6.3 [VDC])	CL05A226MQ5N6J8	Samsung Electro-Mechanics

Table S1. List of components utilized for the construction of implantable oximeter. Table includes a brief description of the component, its part number (as of May of 2025), and the manufacturer that produces the component. Each component was either manually soldered or soldered by the PCB manufacturer used for this study (PCBWay, China).

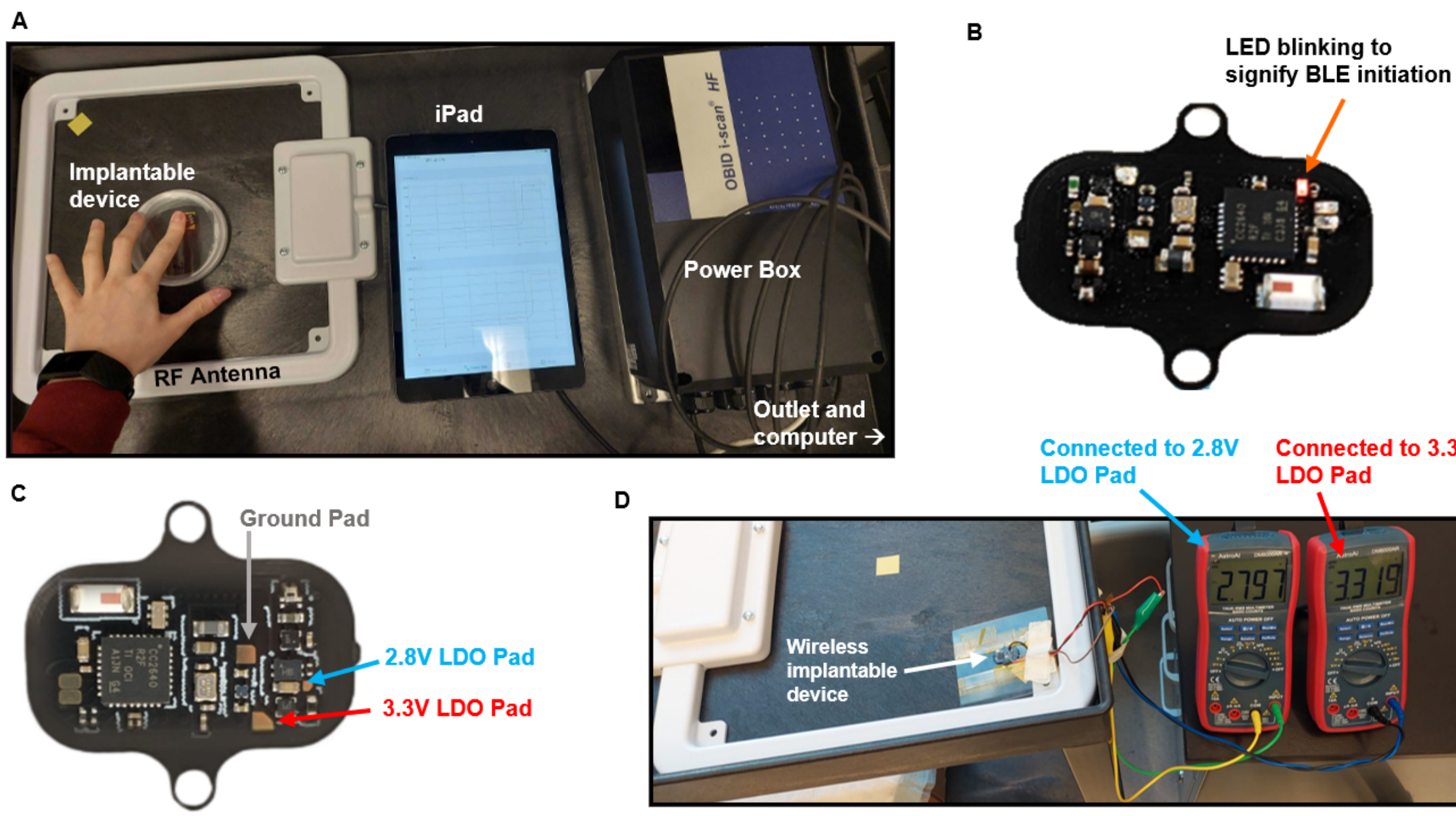

Figure S2. RF Set up and testing of voltage output. **A.** Picture of the devices needed to use the implantable oximeter. The RF setup is composed of a square coil (ID ANT310/310, FEIG Electronics, USA) that connects to a Power Box (ID LR5400, FEIG Electronics, USA) via a CBL wire. The Power Box connects to a computer (not seen in the figure). The RF setup allows for output power control to the implantable device. The tablet (iPad 9th generation, Apple Inc., USA) captures and displays data collection via an in-house application. **B.** A dedicated red LED indicates the end user the status of the device. The blinking of said LED indicates that the implantable device has gone through bootup and is ready to pair to the iPad via Bluetooth. **C.** Picture of oximeter, showcasing the ground pad (black), 3.3 V LDO pad (red), 2.8 LDO pad (blue) are used to probe the voltages and currents of the device at different sections. **D.** Photograph showing testing of oximeter's internal voltages. Two multimeters are connected to the probing pads to test the voltage management of the device. The multimeter on the left shows a reading of 2.797 V, with 2.8 V being an ideal reading. The multimeter on the right shows a reading of 3.319 V, with a 3.3 V being an ideal reading. This testing was performed on all devices prior to parylene coating.

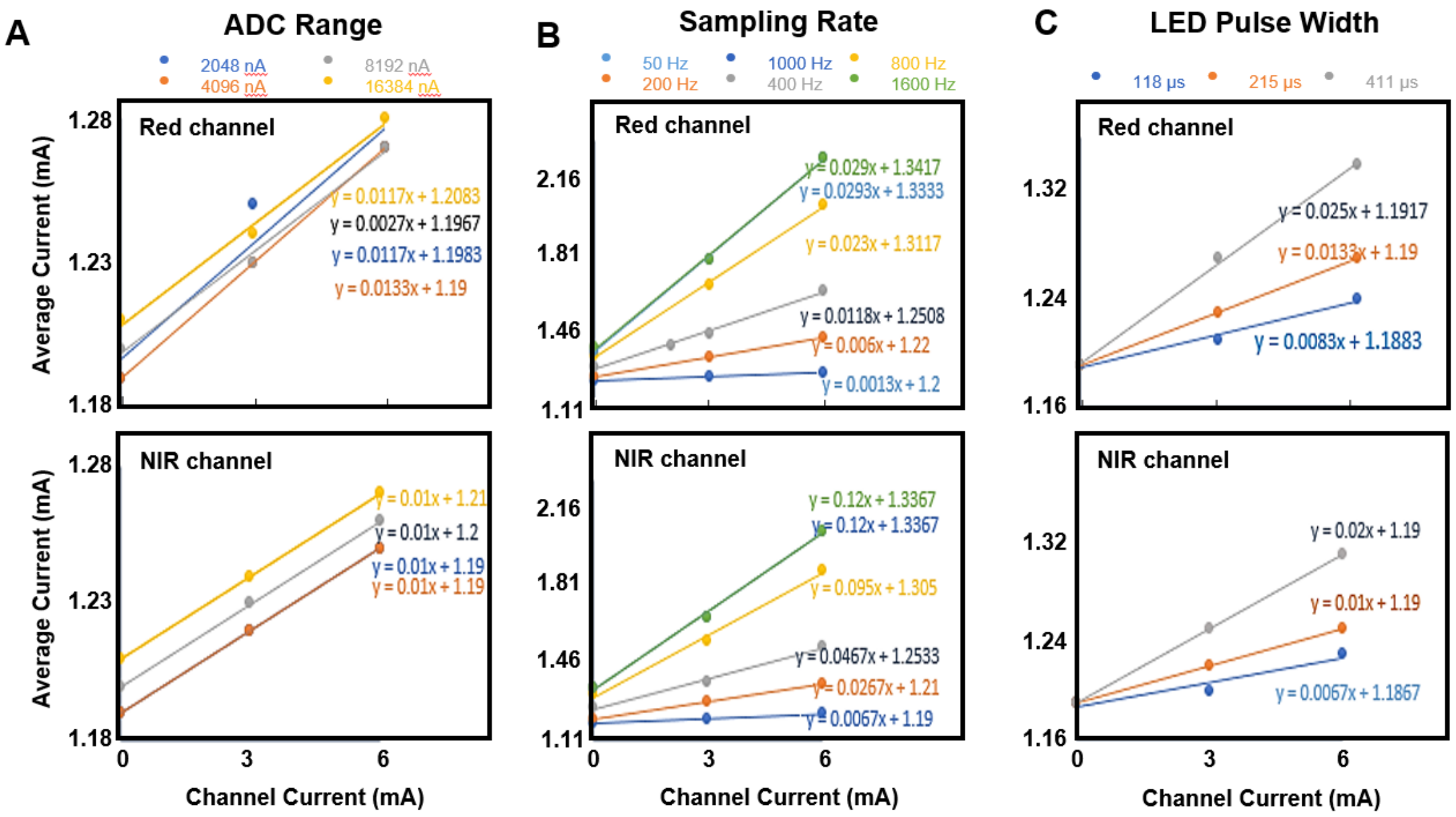

Figure S3. Parameter impact on average current consumption. All measurements were performed using a Nordic Instrument Power Profiler Kit 2 (Nordic Semiconductor, Norway) and its nRF Connect Power Profiler software. Each set of measurements was performed one LED at a time. **A.** The blue, orange, grey, and yellow line represent 2048 nA, 4096 nA, 8192 nA, and 16384 nA, respectively, one line for each of the ADC range options. **B.** The light blue, orange, grey, yellow, navy blue, and green represents 50 Hz, 200 Hz, 400 Hz, 800 Hz, 1000 Hz, and 1600 Hz respectively. **C.** The blue, orange, and grey lines represent 118 µs, 215 µs, and 411 µs, respectively.

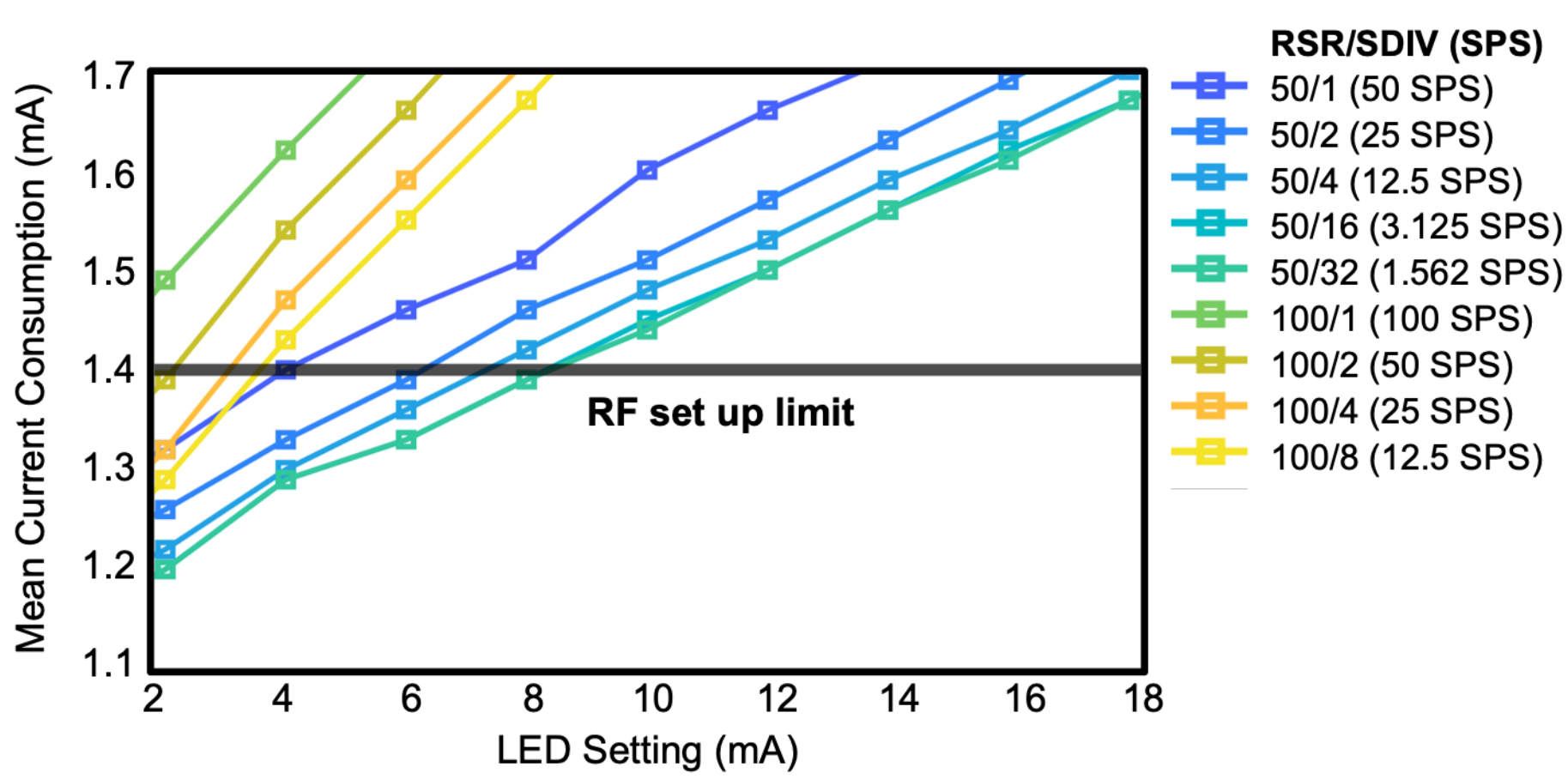

Figure S4. Evaluation of current consumption over different sampling rate configurations and its effect on LED optical output stability. The LEDs were evaluated over different sample rates (SPS), which are determined by two configurations: raw sampling rate (RSR) and sample divider (SDIV). The mean current consumption threshold was empirically determined to be around 1.40 mA. Settings that surpassed this threshold presented different levels of sporadic data drops across LED channels. For this study, mean current consumption was close to threshold to avoid low SNR.

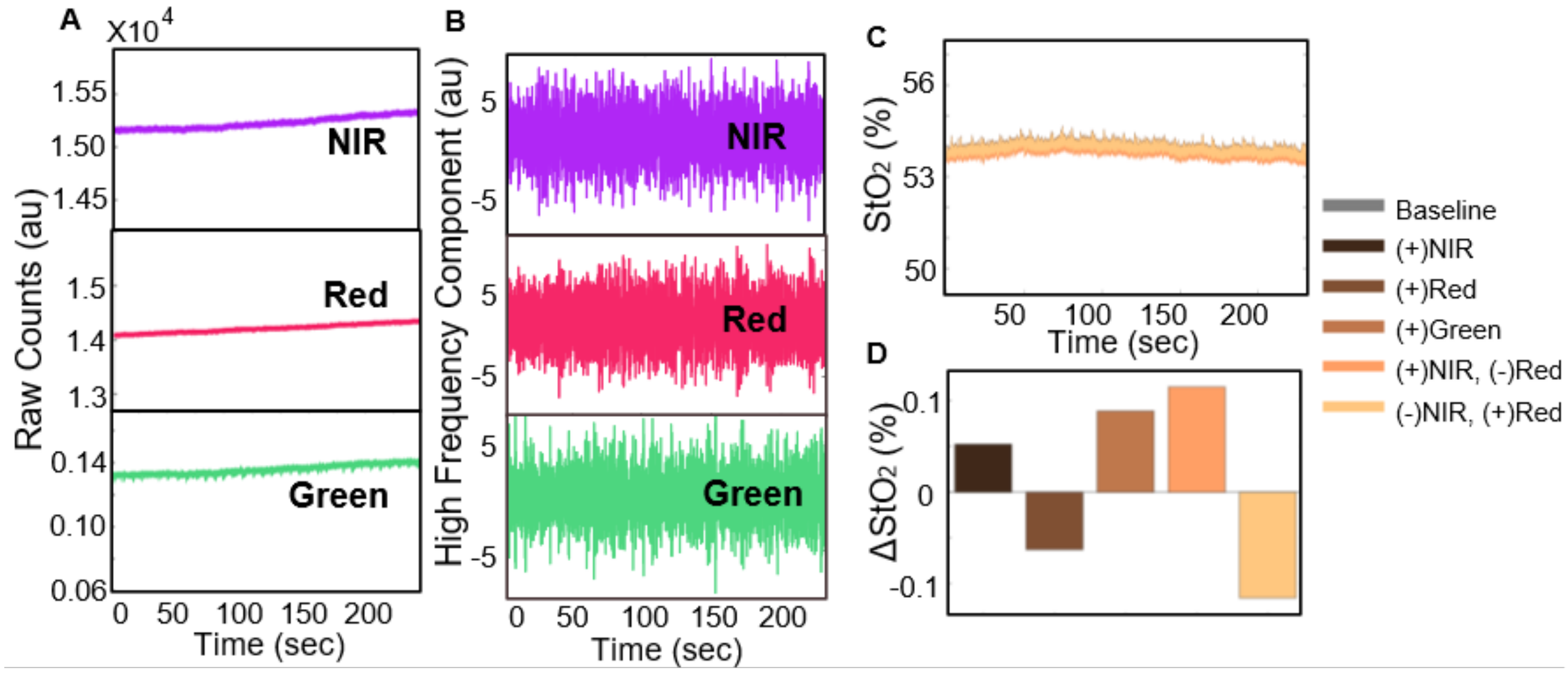

Figure S5. Evaluation of the effects of high frequency noise on the values of tissue oxygenation. A. Raw count values of implantable oximeter during baseline of wake-up study (subdermal implant)**. B.** High frequency noise of the raw counts. **C.** Evaluation of the bias of different combinations of high frequency noise. **D.** Evaluation of difference between bias and ideal tissue oxygenation value for each high frequency noise combination*.*

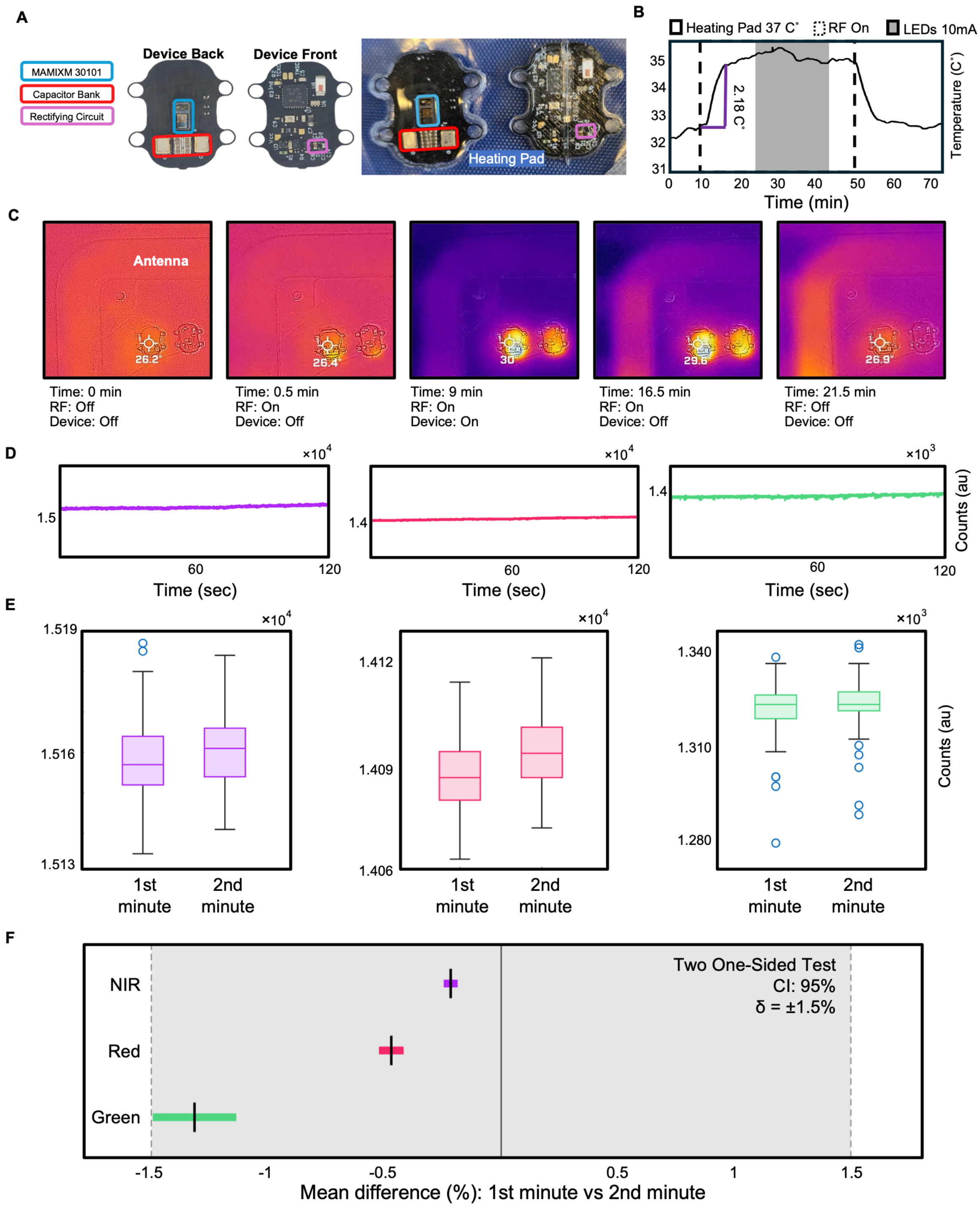

Figure S6. Thermal evaluation of implantable oximeters. A. Visual outline of the oximeter, with its thermally active component highlighted. PDMS-coated devices were placed on top of a heating pad for veterinary use. **B.** Recorded temperature of oximeter during simulated heating for 40 minutes. **C.** Recorded temperatures of oximeter during RF power transmission (up to 21 minutes). **D.** Data channels recordings (880 nm, 660 nm, and 537 nm respectively) during the first two-minutes after implant is turned on. **E.** Comparison of recorded data between the first and second minute for each channel respectively. **F.** Two one-sided test (CI: 95%, δ: 1.5%) analysis between the first and second minute for each data channel recording.

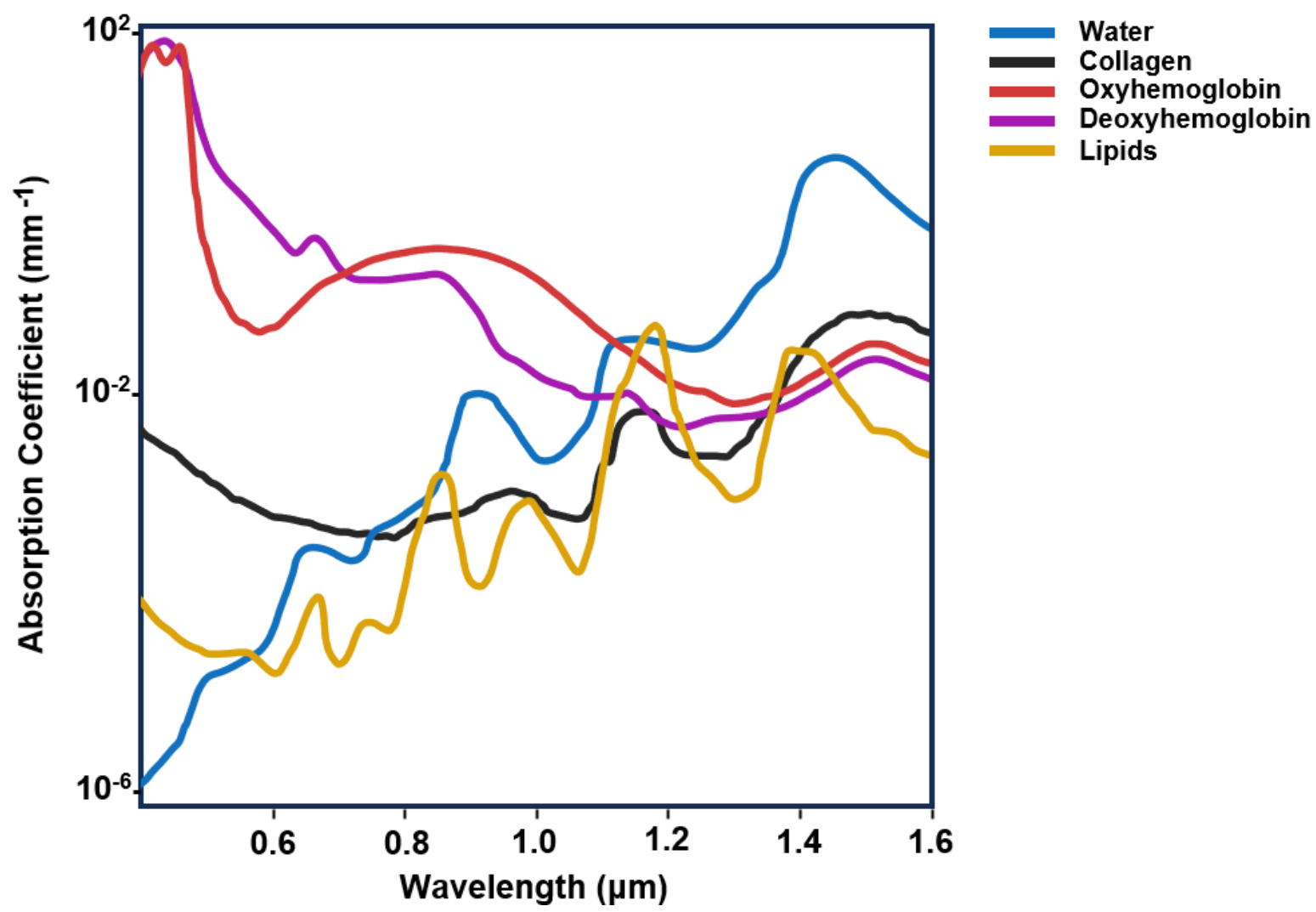

Figure S7. Absorption coefficients of chromophores found in mammals. The data shown in the figure was collected from different sources across literature[1,2].

References

[1] R. Nachabé, D. J. Evers, B. H. W. Hendriks, G. W. Lucassen, M. Van Der Voort, J. Wesseling, T. J. M. Ruers, *Biomed. Opt. Express* **2011**, *2*, 600.

[2] S. K. V. Sekar, I. Bargigia, A. D. Mora, P. Taroni, A. Ruggeri, A. Tosi, A. Pifferi, A. Farina, *J. Biomed. Opt.* **2017**, *22*, 015006.

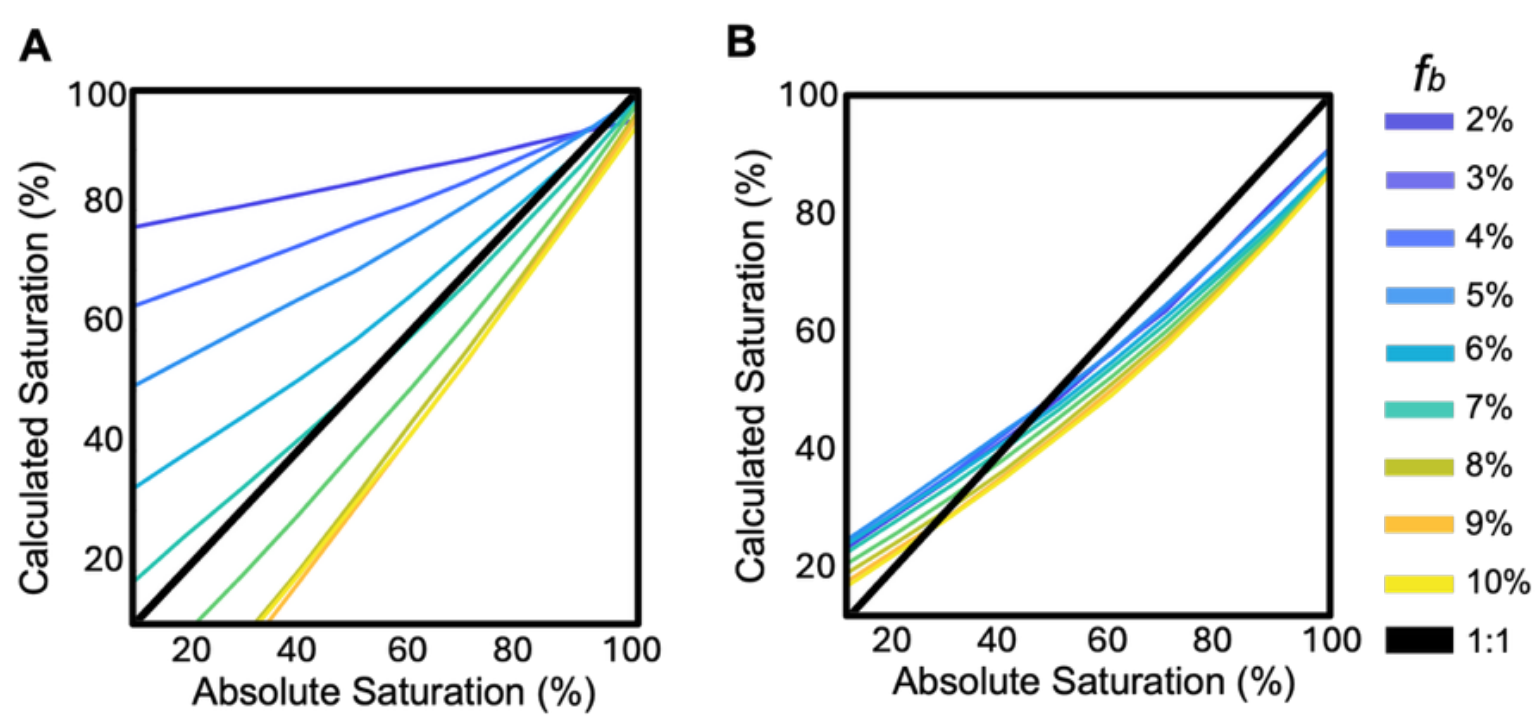

Figure S8. Evaluation of tissue oxygenation over extended blood volume range (2%–10%). A. Calculated tissue oxygen with the two-wavelength classical model, using red (660 nm) and NIR (880 nm) channels. **B.** Calculated tissue oxygen with the three-wavelength model by Nguyen and colleagues, using green (537 nm), red (660 nm), and NIR (880 nm).

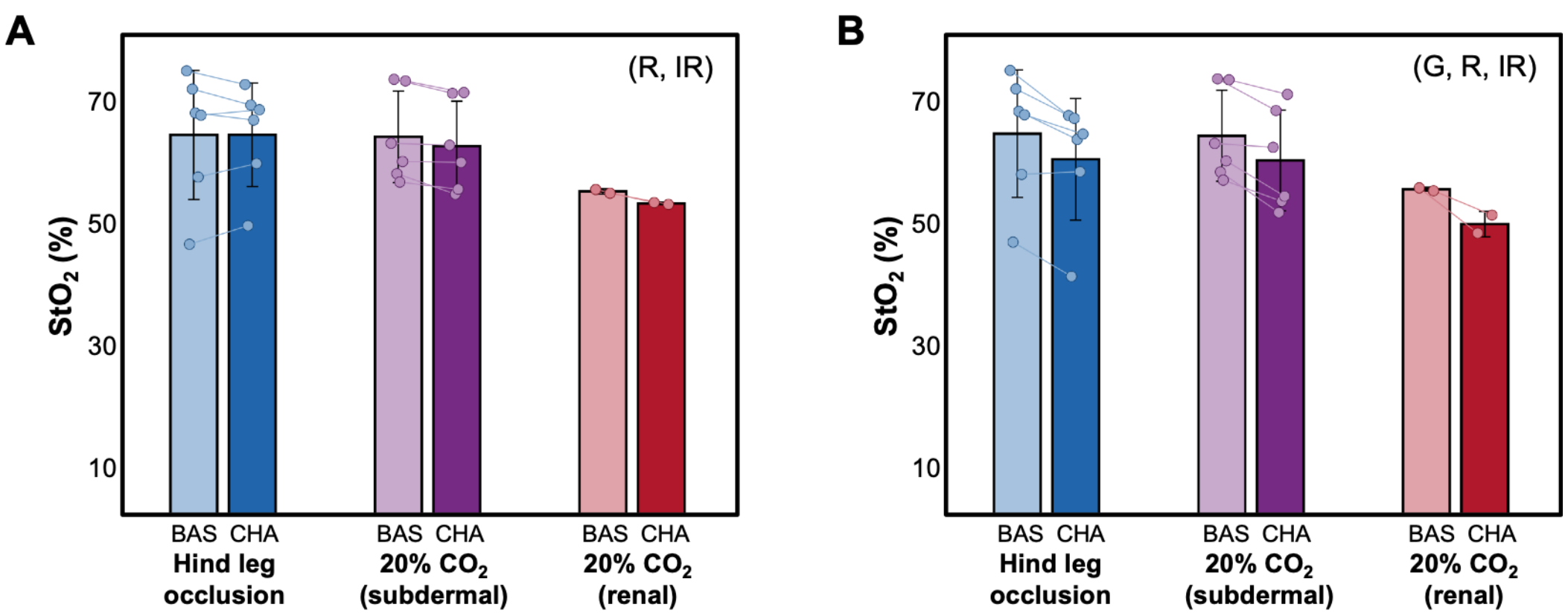

Figure S9. Weekly functionality test results demonstration. Tissue oxygenation means (±1 SD) for baseline (BAS) and stress event (STR) windows (representative 50-second sections) across three trial types. Occlusion (n = 6), subdermal under 20% inhaled CO_2 (n = 6), and renal under 20% inhaled CO_2 (n = 2). **A.** Results from the two-wavelength classical model. **B.** Results from the three-wavelength model by Nguyen and colleagues.

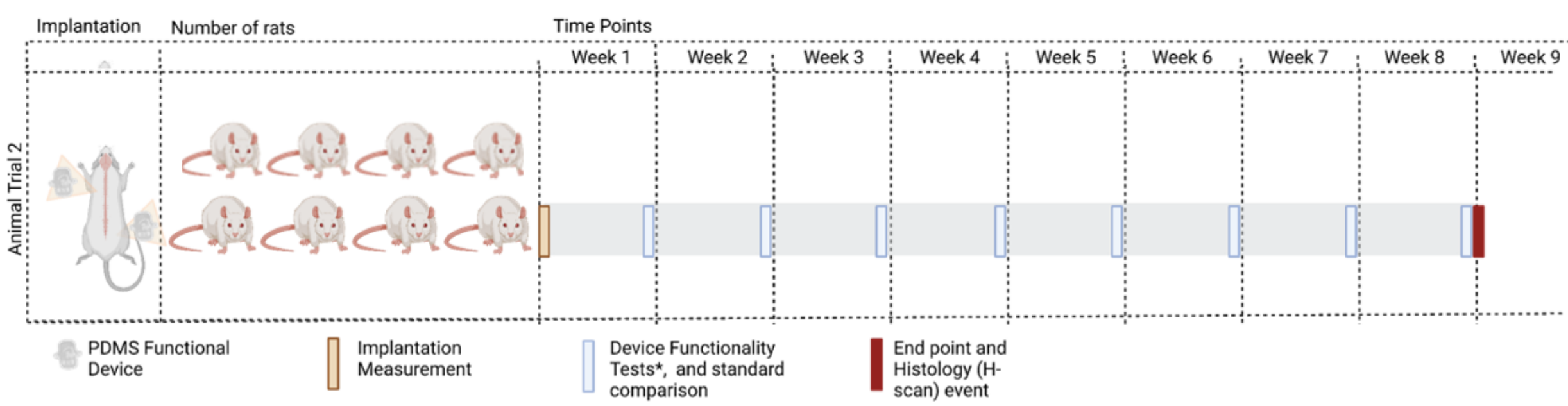

Figure S10. Illustrative figure depicting the timeline structure of the functionality tests performed over the duration of 9 weeks (post-implantation). The functionality test depicted utilized 8 rat models, 4 for of them being female and 4 of the being males. Each rat model got two oximeter devices subdermally implanted in the animals' back (on top of the latissimus dorsi muscles). The devices were located into position using BioGlue (Artivion Inc., USA). Each bar in the figure represents a different event and the grey bar represents the rat model's life span. The yellow bar indicates the day of implantation. Light blue bars indicate functionality tests taking place in which devices cycled through several sets of preset settings (pulse-width, sampling rate, and ADC range). The red bar indicates test endpoint, euthanasia of animal model, and histology collection. A total of 51 functionality settings were tested over the span of this functionality test.

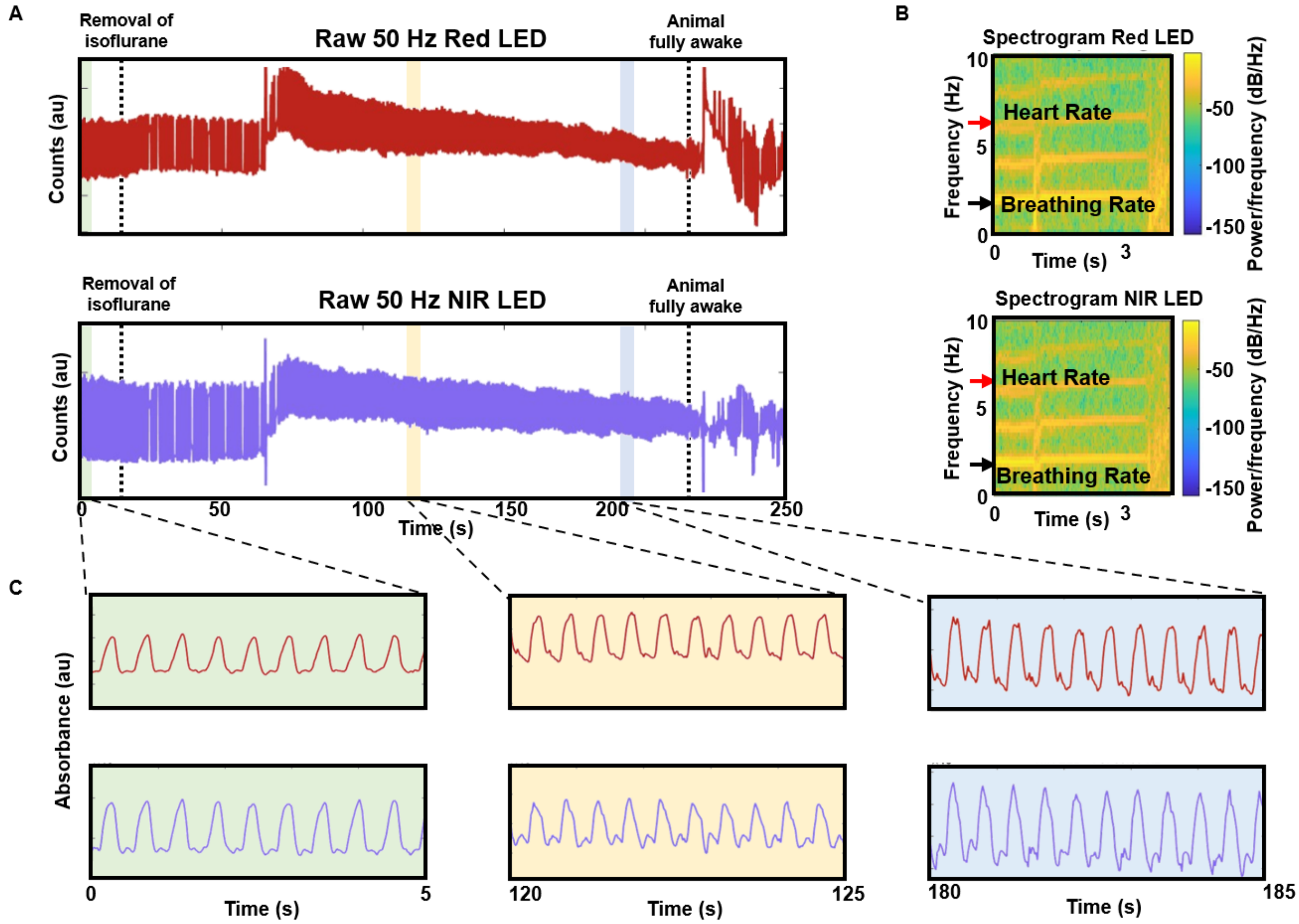

Figure S11. Measurements from an implanted device were taken at 50 Hz (Sample rate: 800 Hz, sample average: 16) as the animal was waking up from 2% isoflurane anesthesia. A. The first 250 seconds of the data collected with a freshly implanted device. The red plot represents reading from the red LED channel, and the purple line represents reading from NIR LED channel. **B.** Spectrogram of the Overall Absorbance Measurement. Top panel corresponds to the red LED channel, and the bottom panel corresponds to NIR LED channel. In both graphs, four distinct lines are seen at approximately the 2 Hz, 4 Hz, 6 Hz, and 8 Hz marks. In both cases all four lines are shifting slightly upwards as time goes on. The line at around the 2 Hz is suggested to be corresponding to breathing rate. The line at around the 6 Hz mark is thought to represent heartrate. **C.** Enlarged raw data plot. These graphs are enlarged versions of the graph found in A of the green, yellow, and blue boxed area. It depicts 5 second snapshots from 0–5 seconds, 120–125 seconds, and 180–185 seconds representing data from when the rat was under isoflurane, when rat was weaning off, and when the rat was almost awake, respectively.

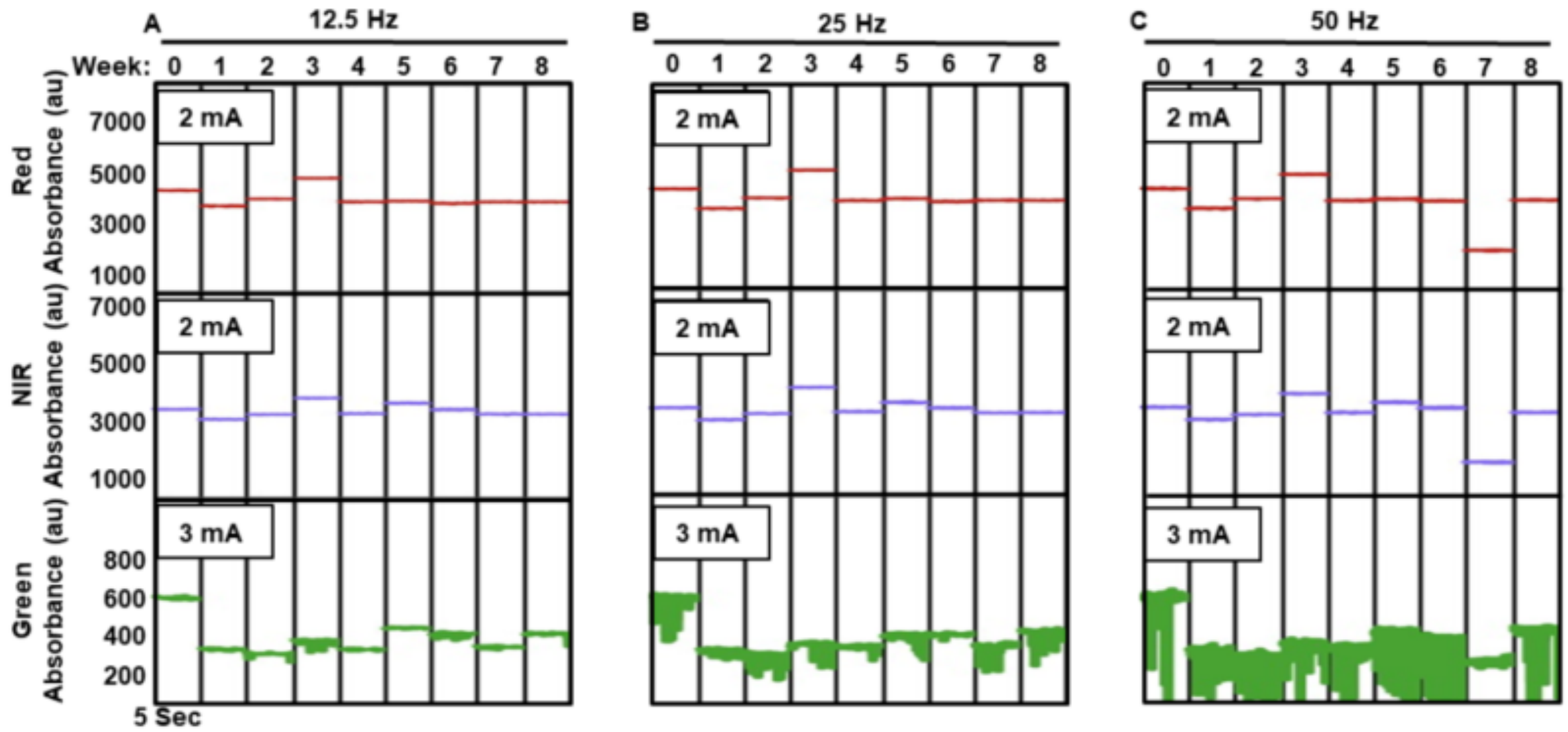

Figure S12. Weekly functionality test results demonstration. The performance of each channel was evaluated each week, totaling 9 weeks from the week of implantation (week zero) to the last week of testing (week 9). Rats were induced with 2% isoflurane and placed in an RF set up. Implanted device would initiate boot up and connect via Bluetooth to an external receiver. Devices would then cycle through a set of 9 settings while data was collected. A–C are displaying 5 second segments of data taken with the red LED (red) set to 2 mA, NIR LED (purple) set to 2 mA, and green (green) LED set to 3 mA. **A**. Weekly data taken at 12.5 Hz. **B**. Weekly data taken at 25 Hz. **C**. Weekly data taken at 50 Hz.

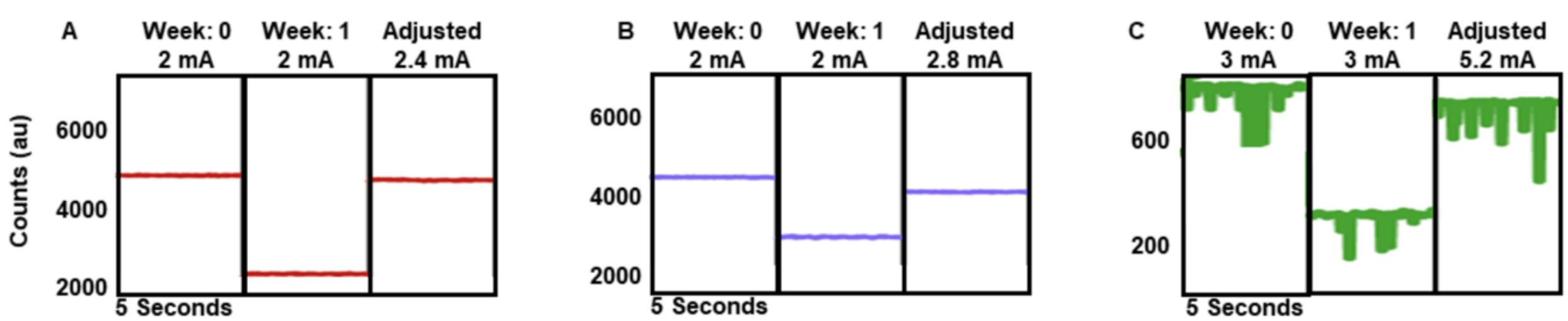

Figure S13. Current adjustability for increased performance in data capture. Five seconds of data were taken at week 0 (left column) and at week 1 (middle and last column), using all LED channels. The first and middle column were set to the same frequency and LED setting but show a drop in counts between week 0 and week 1. The last column displays adjusted data as it was taken with adjusted LED amplitude, increasing the counts measured closer to week 0 counts. **A.** Is the data acquired by the red channel. **B.** Is the data captured by the NIR channel. **C.** Data captured by the green channel.

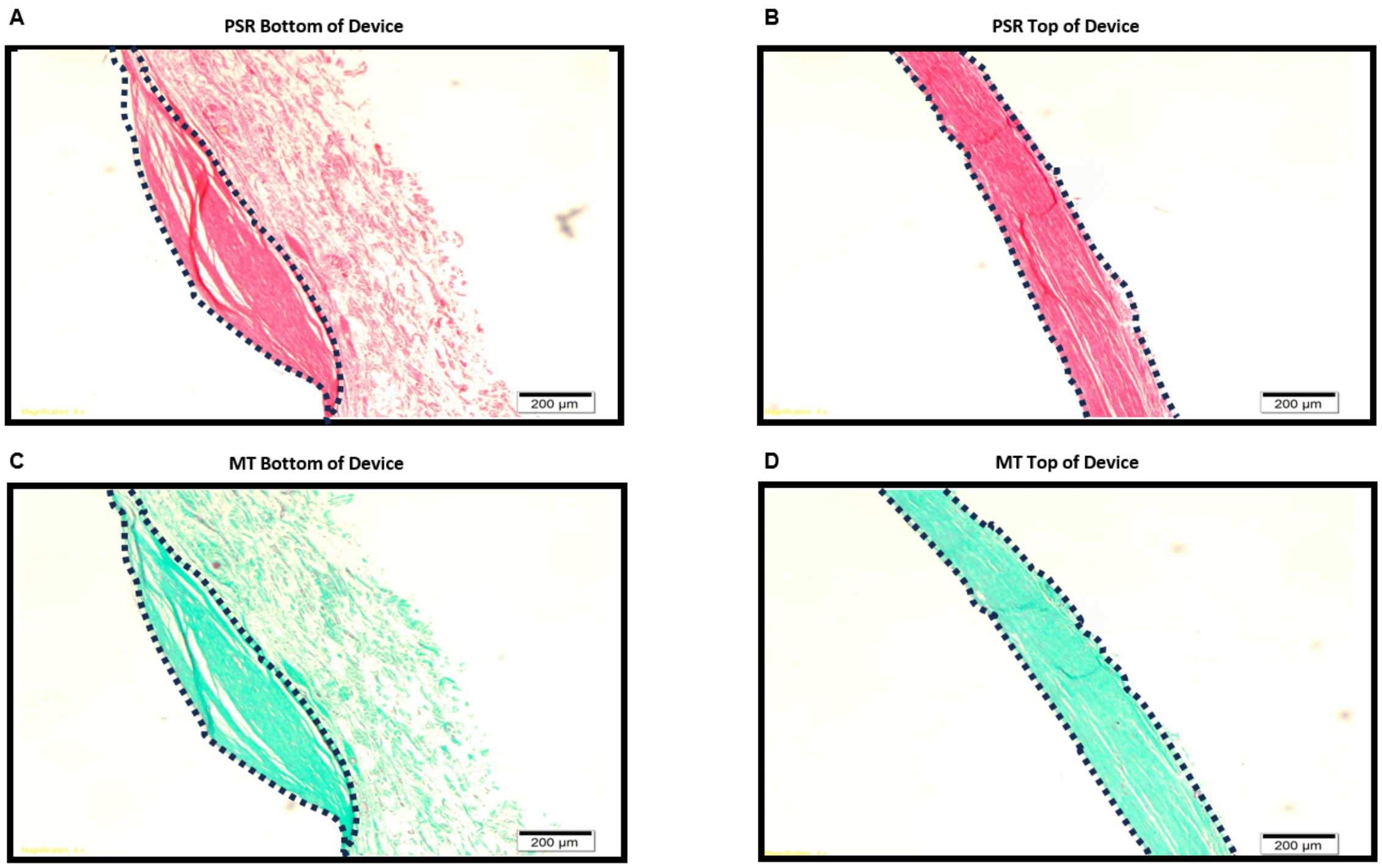

Figure S14. Comparative histological analysis of fibrotic capsule using Picrosirius Red staining (PSR) and Masson's Trichrome staining (MT). For PSR, collagen fibers are stained red, muscle fibers and cytoplasm are stained yellow. For MT, collagen fibers are stained blue/green, cytoplasm and muscle are stained red/pink, and nuclei are stained dark purple/black. Figures **A** and **B** show samples of the bottom (facing muscle) and top (facing skin) sections of the fibrotic capsule, stained with PSR. **C** and **D** depict the bottom (facing muscle) and top (facing skin) samples of the fibrotic capsule, stained with MT. The black outline outlines the boundaries of fibrotic capsule. Images were taken and preprocessed with cellSens Imaging Software (Olympus Corporation, Japan).

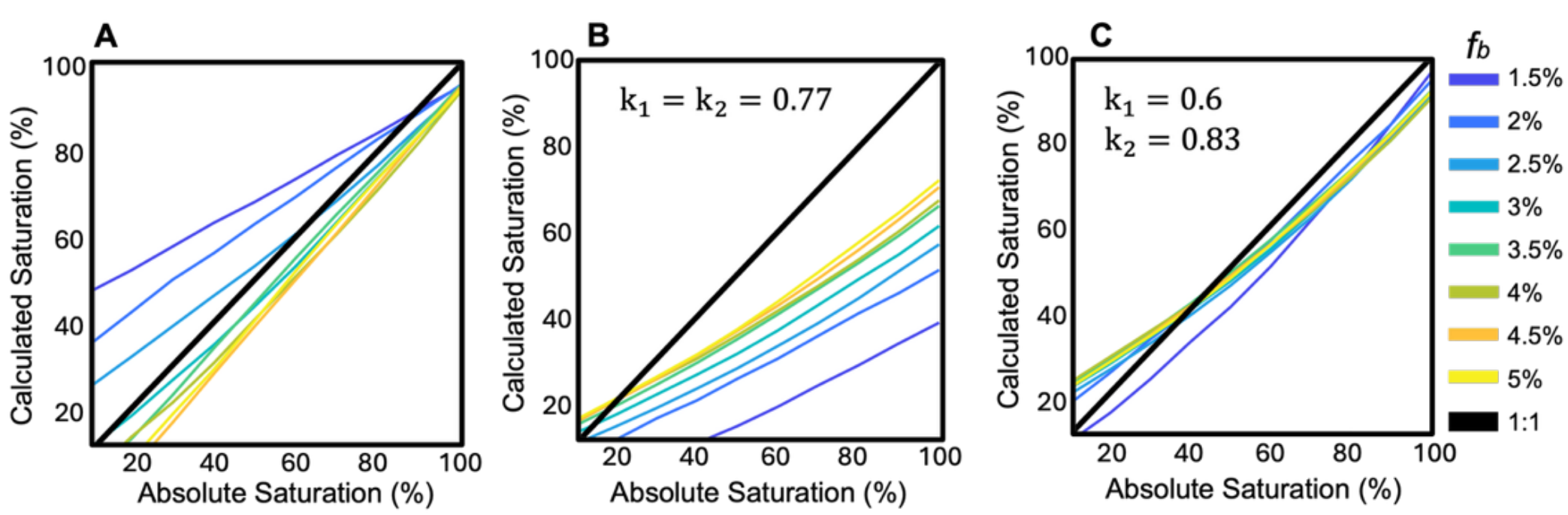

Figure S15. Tissue oxygenation results from simulated muscle layer with added collagen sheet. A. Calculated tissue oxygen with the two-wavelength model. **B.** Calculated tissue oxygen with the three-wavelength model using fitting parameters $k_1 = k_2 = 0.77$. **C.** Calculated tissue oxygen with the three-wavelength model using adjusted fitting parameters of $k_1 = 0.6$ and $k_2 = 0.83$.

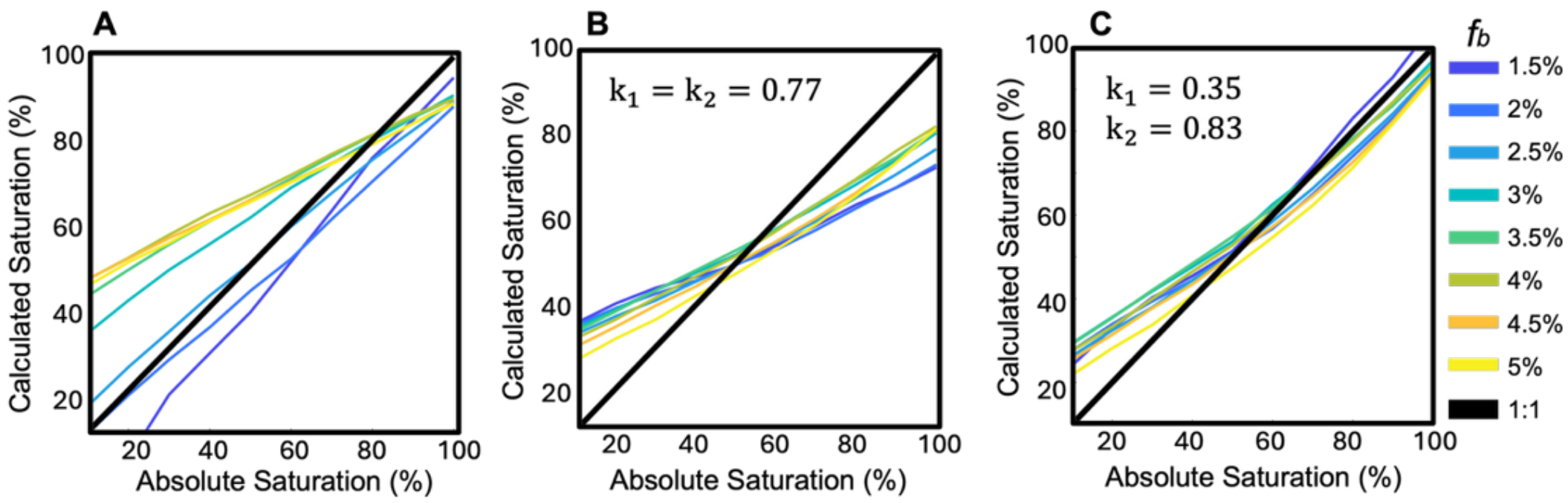

Figure S16. Evaluation of tissue oxygen estimation over increased source-detector separation distance. A. Calculated tissue oxygen with the two-wavelength model. **B.** Calculated tissue oxygen with the three-wavelength model using fitting parameters $k_1 = k_2 = 0.77$. **C.** Calculated tissue oxygen with the three-wavelength model using adjusted fitting parameters of $k_1 = 0.35$ and $k_2 = 0.83$.
